# Cow’s microbiome from antepartum to postpartum: A long-term study covering two physiological challenges

**DOI:** 10.3389/fmicb.2022.1000750

**Published:** 2022-11-16

**Authors:** Johanna Tröscher-Mußotter, Simon Deusch, Daniel Borda-Molina, Jana Frahm, Sven Dänicke, Amélia Camarinha-Silva, Korinna Huber, Jana Seifert

**Affiliations:** ^1^HoLMiR—Hohenheim Center for Livestock Microbiome Research, University of Hohenheim, Stuttgart, Germany; ^2^Institute of Animal Science, University of Hohenheim, Stuttgart, Germany; ^3^Institute of Animal Nutrition, Friedrich-Loeffler-Institut, Federal Research Institute for Animal Health, Braunschweig, Germany

**Keywords:** calving, dairy cow, duodenum, LPS, metabolome, microbiome, rumen, transition

## Abstract

Little is known about the interplay between the ruminant microbiome and the host during challenging events. This long-term study investigated the ruminal and duodenal microbiome and metabolites during calving as an individual challenge and a lipopolysaccharide-induced systemic inflammation as a standardized challenge. Strong inter- and intra-individual microbiome changes were noted during the entire trial period of 168 days and between the 12 sampling time points. *Bifidobacterium* increased significantly at 3 days after calving. Both challenges increased the intestinal abundance of fiber-associated taxa, e.g., *Butyrivibrio* and unclassified *Ruminococcaceae*. NMR analyses of rumen and duodenum samples identified up to 60 metabolites out of which fatty and amino acids, amines, and urea varied in concentrations triggered by the two challenges. Correlation analyses between these parameters indicated a close connection and dependency of the microbiome with its host. It turns out that the combination of phylogenetic with metabolite information supports the understanding of the true scenario in the forestomach system. The individual stages of the production cycle in dairy cows reveal specific criteria for the interaction pattern between microbial functions and host responses.

## Introduction

The modern dairy cow is confronted with a multitude of challenges. This, among others, includes feed changes, pathogens, heat stress, and the transition from dry period to high-performance milk production. These impair the cow’s health and hence, productivity as well as product quality. Ketosis, metritis, milk fever, and displaced abomasum might go along with or even originate from a disrupted forestomach and gut microbial community and might appear as a decrease in microbial diversity, richness, and functionality (Pitta et al., 2014; Khafipour et al., 2016). The gut-brain-axis, the mitochondria-microbiota-intertalk (Saint-Georges-Chaumet and Edeas, 2015), and the entero-mammary pathway (Rainard, 2017) are studied intensively, more and more revealing the importance of the microbiome in steering the host metabolism. Nevertheless, since modern animal production requires distinctively different physiological features than those established by evolution over thousands of years, the animals’ health is mostly affected negatively, as milk yields continuously increase. Hence, shedding more light on the complex metabolite cross-talk between host and microbes could bear the potential to improve animal welfare and thereby also have crucial economic impacts as medical expenses can be a major burden for animal farmers (Schlau et al., 2012).

The transition from the dry period to lactation is one of the most critical phases in a cows’ life. The metabolism focuses on milk production with high energy and protein requirements at limited energy intake, making the cow more susceptible to internal and external challenges and often leading to inflammatory conditions (Bradford et al., 2015). The requirement of energy increases within hours after calving due to the onset of lactation, which can only be met by enhanced proportions of concentrate feed containing mostly rapidly fermentable carbohydrates. This, in turn, might adversely affect rumination activity and consequently, ruminal and duodenal microbiota. This sudden regimen shift was not anticipated by nature since calving would be during early summer, with ample and steady fresh forage availability. The shift toward intake of rapidly fermentable carbohydrates with insufficient physically effective fiber might trigger a vicious cycle. The subsequent reduction in alkaline saliva production (Yang and Beauchemin, 2006) fails to buffer the drop in rumen pH, due to higher ruminal lactate and short-chain fatty acid (SCFA) production, leading to an increase of Gram-negative bacteria rich in membrane lipopolysaccharides (LPS; Khafipour et al., 2009; Saleem et al., 2012). The shift in dietary composition may also affect the intestinal epithelial lining’s permeability, which can represent the gateway for pathogenic organisms and LPS influxes to the blood, causing inflammatory host conditions as the immune system reacts to the antigens (Baumgard and Rhoads, 2013; Schroeder et al., 2018). This impaired health status is often accompanied by secondary symptoms such as fever, lowered feed intake, and milk yield, as well as declined rumination activity (Baumgard and Rhoads, 2013). The severity of these conditions might vary largely among individuals despite the equal feeding regimen (Bradford et al., 2015) and may derive from varying individual metabolic or energetic capabilities (Jami et al., 2013). Cellular energy derives from the mitochondria, whereby their vitality is assumed to play a key role in health and disease of the organism (Duchen, 2004). For mitochondrial energy conversion, L-carnitine plays a crucial role as carrying long-chain fatty acids as acylcarnitines, into the mitochondrial matrix, where they are further included to β-oxidation. In previous studies, cows with higher blood serum acylcarnitines had extended productive lifespans compared to those exiting earlier (Huber et al., 2016). The supplementation of this metabolite may hence aid the dairy cow through this critical phase.

It is still unclear, which factors contribute to the robustness of individual cows, and how they manage the balance between high productivity at good fitness and therefore contribute to high farm profitability. The response of the microbiome and its possible function toward stimulating the host physiology and metabolism during host challenging situations are also less studied so far. Therefore, scoping into the rumen as the major site of energy conversion and the duodenum as the first site of host nutrient uptake could enlighten these responses to challenging periods.

This study hypothesizes that modern dairy cows respond to the challenge of calving, the subsequent feed change, and a LPS-induced standardized inflammation by dynamic adaptation of ruminal and duodenal bacteriomes. These responses either support or intensify the severity of the reaction toward stress. Additionally, the role of supplemented L-carnitine for the bacterial consortium in the matrixes, rumen, and duodenal fluid, across a particularly long period of time, is elucidated. This study connects detected metabolites with bacterial abundances and selected host health parameters to pinpoint possible interactions.

## Results

**Figure 1:**
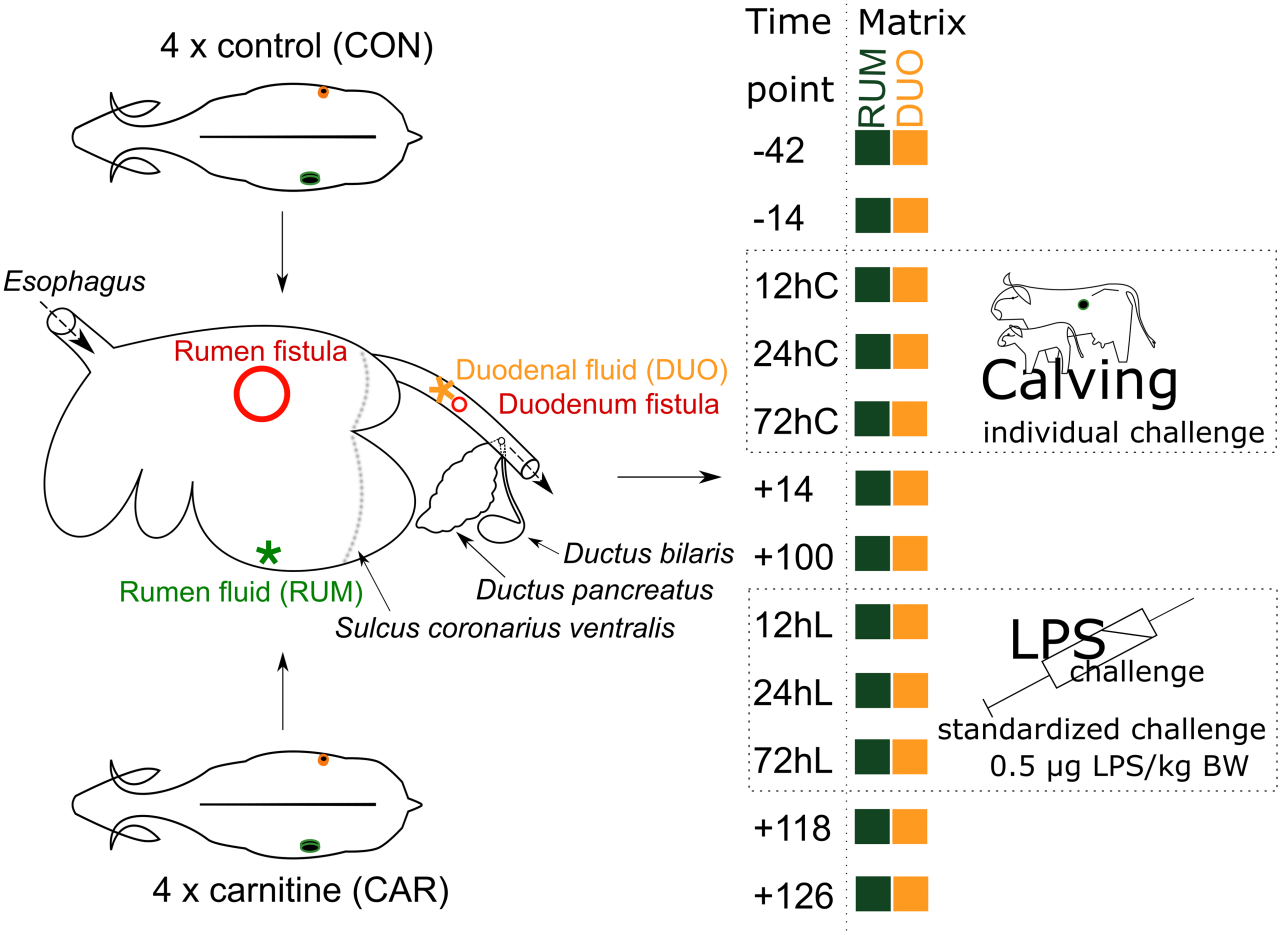
MitoCow—Trial setup. Rumen (RUM) and duodenal (DUO) fluid samples were taken from 4 control and 4 carnitine supplemented animals at 12 different time points. Stars indicate where the samples were taken from red circles indicate the place of fistulation. Time points including a “–“or “+” indicate days *antepartum* or *postpartum* and time points including “hC” or “hL” are samples taken at 12, 24, or 72 h after calving or LPS challenge, respectively.

The animal trial covered 168 days and used eight double fistulated dairy cows, which resulted in a total of 143 samples from rumen (RUM) and duodenum fluid (DUO; Figure 1). From −42 to +14 pH in RUM dropped significantly (Wilcoxon test *p* = 0.009) from an average of 6.9 ± 0.3 (SD) to 5.8 ± 0.6 (Supplementary Figure 1). This significant difference was still observed after feed adaptation of the bacteriome until +100 (*p =* 0.03). LPS challenge significantly increased pH in RUM and DUO (+100 vs. 12hL, both *p* ≤ 0.04). The pH values in DUO increased from −14 (2.4 ± 0.2) to shortly after calving (12hC, pH 3.2 ± 0.3; 72hC, pH 2.9 ± 0.4 both *p* = 0.02).

### Temporal evolvement of the microbiota in rumen and duodenum

Operational taxonomy units (OTUs) of 68 RUM and 75 DUO samples were taxonomically assigned to 16 phyla, 65 families, and 102 genera. Bacteroidetes [average (av.): RUM = 51% ± 13 (SD), DUO = 40% ± 12; *p* < 0.0001], Firmicutes (RUM = 28% ± 11, DUO = 39% ± 12; *p* < 0.0001), and Actinobacteria (RUM = 7% ± 9, DUO = 8% ± 9; not significant) were the dominant phyla in RUM and DUO samples (Supplementary Tables 1, 2).

**Figure 2:**
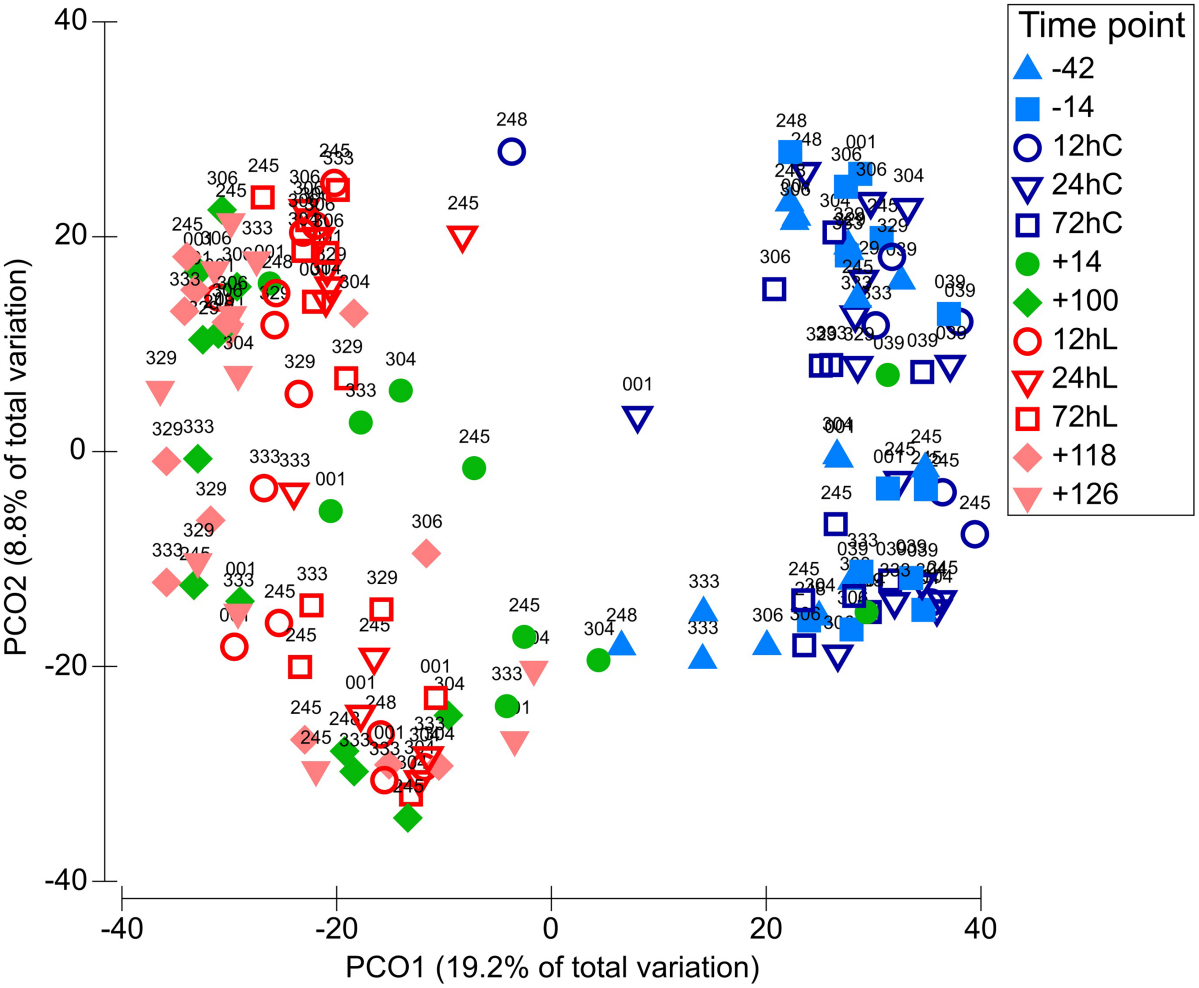
Variation of the bacteriome along the full sampling period. PCO plot on OTU level including 143 samples grouped among 12 time points. Matrix definitions are neglected. Time points including a “–” or “+” indicate days *antepartum* or *postpartum* and time points including “hC” or “hL” are samples taken at 12, 24, or 72 h after calving or LPS challenge, respectively.

The L-carnitine supplementation was not affecting bacterial communities in both matrixes (Supplementary Table 1, Supplementary Figures 2A,B); therefore, CON and CAR sequencing results will be combined from now on. RUM and DUO showed similar bacterial community compositions throughout the 168 days of sampling (Supplementary Figure 3A), except at −42 and −14, which revealed significant differences (ANOSIM *p* ≤ 0.05, Supplementary Table 2). Due to the high degree of similarity, the microbiota of both matrixes are not distinguished in the following. A clear separation along time points can be seen, especially between *ap* and *pp* time points (Figure 2; Supplementary Figure 3B). RUM −42 was significantly different from RUM 72hC, 12hL, 72hL, +118, and +126 (ANOSIM all *p* = 0.0003, Supplementary Table 3), due to higher abundance of *Ruminobacter* and *F. succinogenes* in RUM −42 samples (SIMPER analysis). DUO samples at −42 were significantly different from +100, 12hL, 24hL, +118, and +126, respectively (ANOSIM all *p* ≤ 0.0003) due to higher abundances of *Succiniclasticum* and *Ruminobacter* (SIMPER analysis).

**Figure 3:**
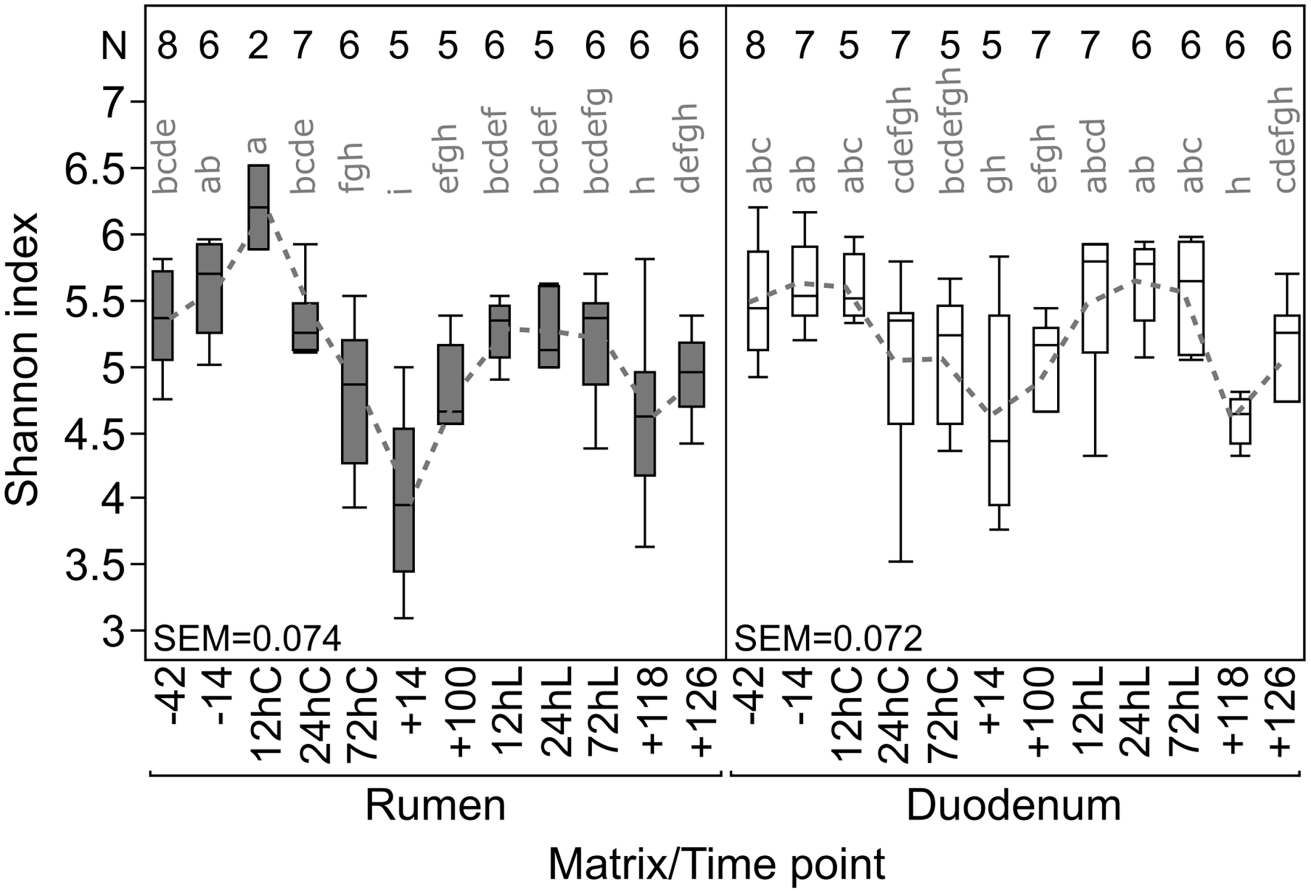
Individual Shannon diversity index throughout the complete trial period for rumen and duodenal fluid samples. Time points including a “–” or “+” indicate days *antepartum* or *postpartum* and time points including “hC” or “hL” are samples taken at 12, 24, or 72 h after calving or LPS challenge, respectively. *N*-Values refer to total samples included per time point.

Shannon diversity (α-diversity) varied throughout the entire trial period on average between 4.0 (+14) to 6.2 (12hC) in RUM and 4.4 (+14) to 5.8 (12hL) in DUO, demonstrating slightly greater diversity fluctuations in RUM (Figure 3). Diversity significantly decreased in both matrixes from −14 to +14 (RUM: *p* = 0.008, DUO: *p* = 0.05) and increased, induced by LPS challenge (+100 vs. 24hL, RUM: *p* = 0.06 n.s., DUO: *p* = 0.02). Significant negative correlations were observed between α-diversities and abundances of certain bacterial taxa such as *Olsenella*, unclassified *Lachnospiraceae* and *Roseburia* abundances in both sample types (Supplementary Table 3).

### Temporal and individual variations of bacterial groups

**Figure 4:**
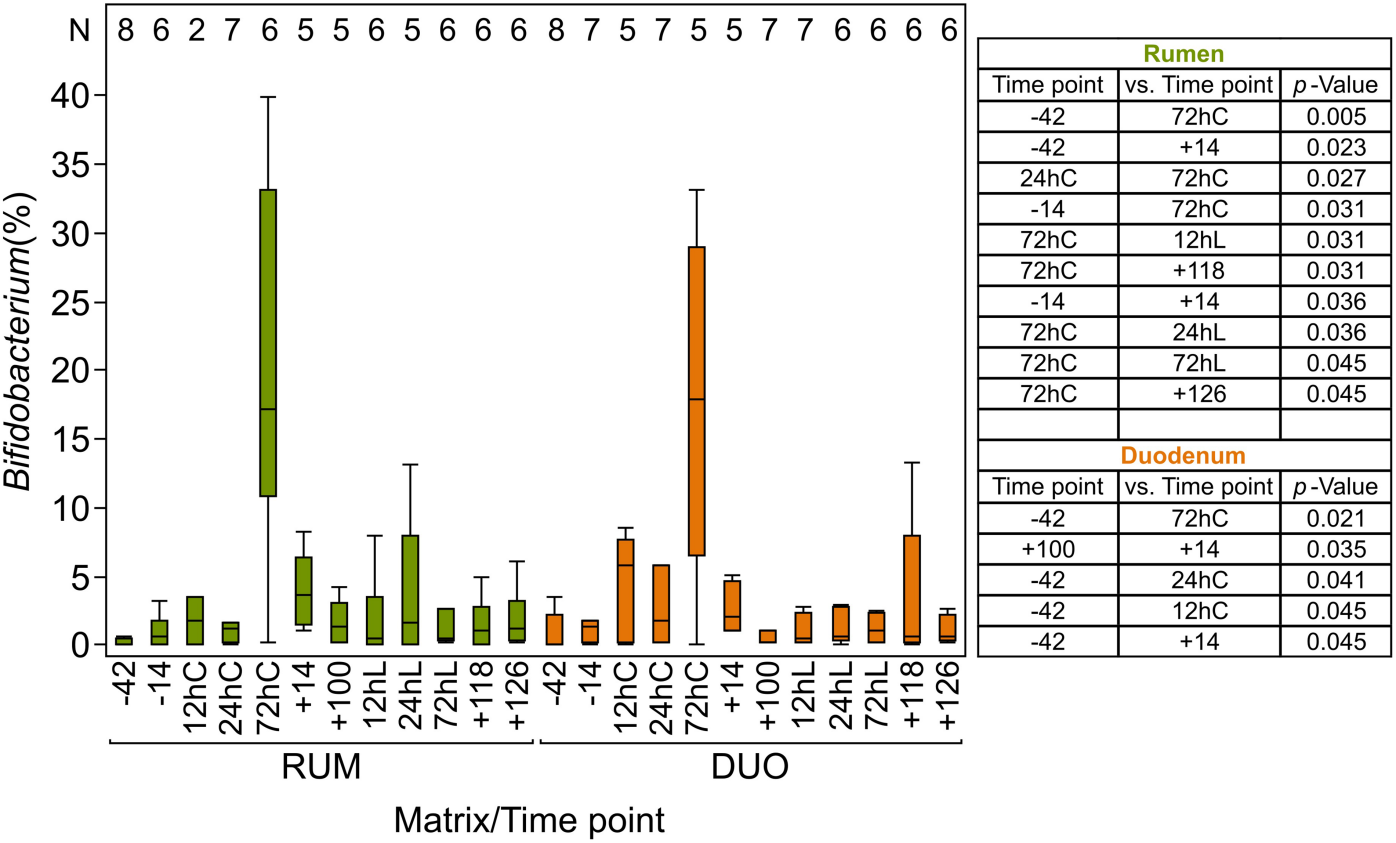
Relative abundance (%) of *Bifidobacterium* in rumen and duodenal fluid samples The table includes significantly different time points (*p* ≤ 0.05) among rumen and duodenal fluid samples, calculated using non-parametric Wilcoxon test. Time points including a “–” or “+” indicate days *antepartum* or *postpartum* and time points including “hC” or “hL” are samples taken at 12, 24, or 72 h after calving or LPS challenge, respectively. *N*-Values refer to total samples included per time point.

The overall taxonomic composition showed fluctuations in abundances in both sampling sites along the animal trial caused by calving, changes in feeding regime, and LPS challenge. Abundances of *Bifidobacterium* increased significantly at 72hC in both matrixes (RUM = 20% Wilcoxon test *p* ≤ 0.005; DUO = 18% *p* ≤ 0.02; Figure 4) compared to −42 (RUM, DUO = both 1%) and were also significantly higher in DUO at +14 vs. +100 (*p* ≤ 0.03). Thereafter, *Bifidobacterium* abundance decreased to *antepartum* (*ap*) abundance level*. Olsenella* species increased significantly after calving and feed adaptation in RUM (24hC: 1% vs. +14: 9%, *p* = 0.03) and DUO (24hC: 0.4% vs. +14: 8%, *p* = 0.009). At LPS challenge time points, a decrease in abundance was observed, which was significant in RUM (+100: 5% vs. 72hL: 2%, *p* = 0.02).

Unclassified *Clostridiales* abundances significantly decreased in DUO after calving and feed change (−14 to 24hC, *p* = 0.03) and significantly increased shortly after the LPS challenge (+100 to 12hL, *p* = 0.02). Unclassified *Ruminococcaceae* increased significantly with calving and/or feed change in both matrixes (RUM: −42 to 72hC, *p* < 0.02; DUO: −42 to 12hC *p* = 0.007) and in DUO samples during LPS challenge (+100 to 72hL, *p* < 0.02). Unclassified *Lachnospiraceae* increased in RUM significantly with the calving and/or feed-changing event (−14 to +14, *p* = 0.04). *Fibrobacter* was significantly higher abundant in DUO than in RUM (*p* = 0.02). *Butyrivibrio* in both matrixes significantly decreased with calving and/or feed change (RUM: 24hC vs. +14, *p* = 0.03; DUO: −14 vs. +14, *p* = 0.009) and significantly increased with LPS challenge in DUO (+100 vs. 24hL, *p* = 0.02).

**Figure 5:**
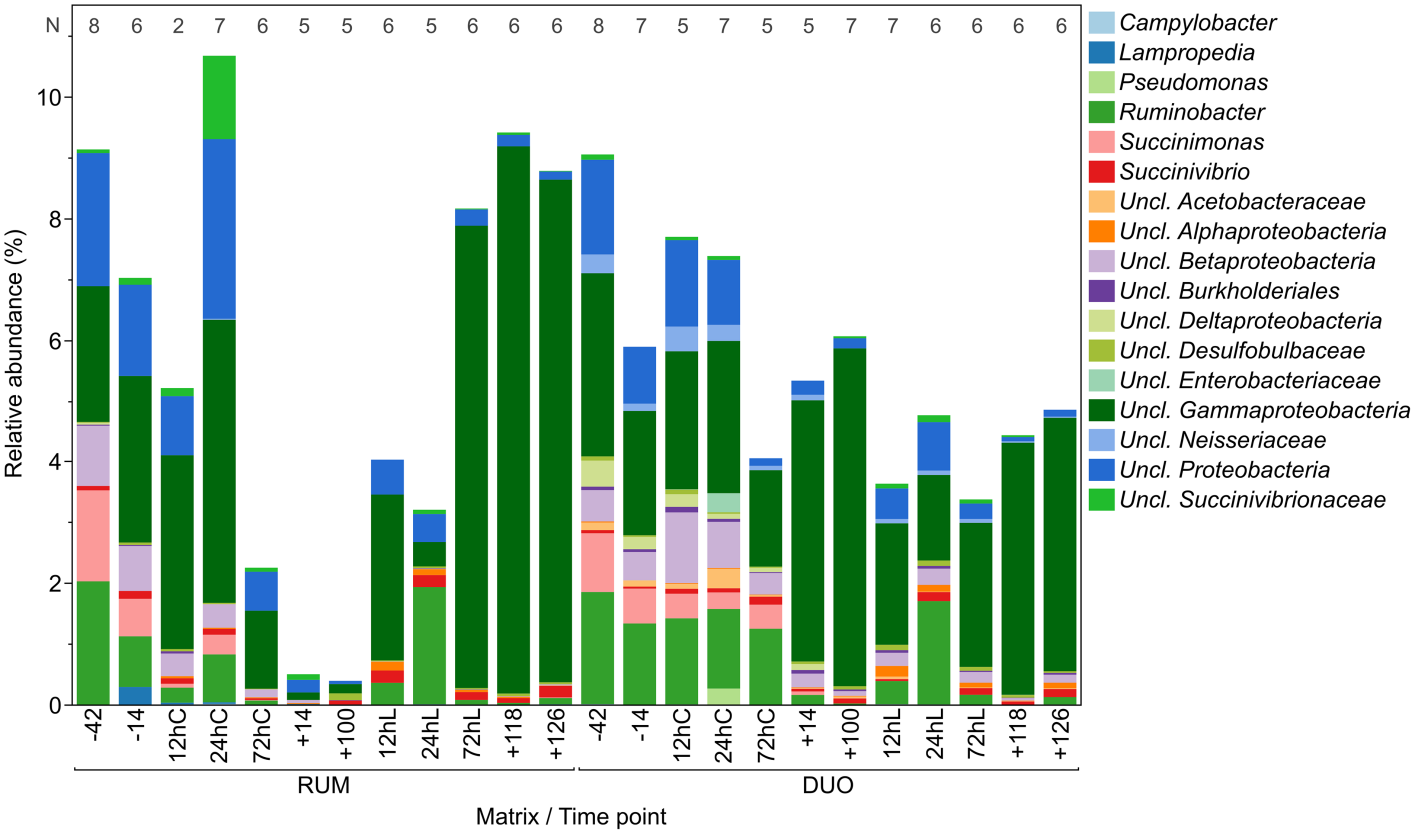
Relative abundance of Proteobacteria genera in rumen (RUM) and duodenal fluid (DUO). *N*-Values refer to sample number included per time point. Time points including a “–” or “+” indicate days *antepartum* or *postpartum* and time points including “hC” or “hL” are samples taken at 12, 24, or 72  h after calving or LPS challenge, respectively.

Proteobacteria showed similar abundances in RUM and DUO and higher genera diversity and evenness before calving and feed change. This phylum significantly decreased after calving and feed change (−14 vs. +14, +100, *p* = 0.008, Figure 5) and increased significantly during LPS challenge (+100 vs. 12hL, *p* = 0.01). After calving and feed change, unclassified *Gammaproteobacteria* clearly dominated this phylum in DUO which was not the case in RUM samples. Here, the LPS challenge triggered a very clear increase of unclassified *Gammaproteobacteria*. This genus dominated the total sequenced Proteobacteria at the end of the trial, summing more than 95% in RUM and 85% in DUO. In RUM, a significant decrease of *Ruminobacter* is observed from −42 to +14 (*p* = 0.02) and an increase induced by the LPS challenge (+14 vs. 72hL, *p* = 0.02). In both matrixes, *Succinimonas* completely vanished after feed calving and feed change (−42 vs. +14 − +126, RUM: *p* ≤ 0.02, DUO: *p* ≤ 0.007). RUM *Succinivibrio* significantly decreased from −14 to +14 (*p* ≤ 0.05) but recovered from +100 on.

### Variations of rumen and duodenal metabolites

**Figure 6:**
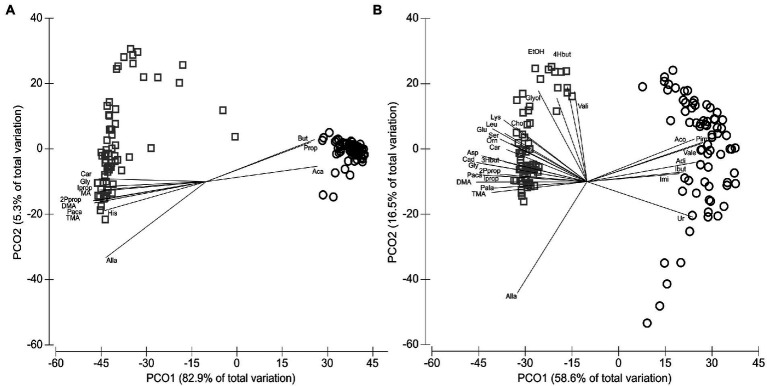
Distribution of metabolites identified by NMR analysis. **(A)** Metabolites of the animal’s rumen fluid (RUM, circles, *N* = 71) and duodenal fluid (DUO, squares, *N* = 67) samples including Spearman’s correlation vector (Spearman’s *r* ≥ 0.85). **(B)** Metabolites of rumen (circles) and duodenal fluid (squares) samples including Spearman’s correlation vector (*r* ≥ 0.65) and excluding acetate, propionate and butyrate. 2Pprop, 2-Phenylpropionate; 3Hbut, 3-Hydroxybutyrate; 4Hbut, 4-Hydroxybutyrate; Aca, Acetate; Aco, Acetone; Adi, Adipate; Alla, Allantoin; Asp, Aspartate; But, Butyrate; Cad, Cadaverine; Car, Carnitine; Cho, Choline; DMA, Dimethylamine; EtOH, Ethanol; Glut, Glutamate; Gly, Glycine; Glyol, Glycerol; Ibut, Isobutyrate; Imi, Imidazole; Iprop, Isopropanol; Leu, Leucine; Lys, Lysine; Orn, Ornithine; Paca, Phenylacetate; Pala, Phenylalanine; Pim, Pimelate; Ser, Serine; TMA, Trimethylamine; Ur, Urea; Vale, Valerate; Vali, Valine.

In total, 60 host and bacteria-derived metabolites were measured using NMR spectroscopy (Supplementary Table 4). RUM (*N* = 71) and DUO (*N* = 67) samples were significantly grouping apart from each other (ANOSIM *p* = 0.0001; Global-R = 0.996; Figure 6A; SIMPER 81.1% av. dissimilarity between RUM and DUO) with RUM samples clustering closer together (85.5% av. similarity) compared to DUO samples (67.2% av. similarity). The pairwise correlation analyses between metabolites of both matrixes can be taken from Supplementary Table 5. Spearman’s correlation vectors with *r* ≥ 0.85 indicated a strong, significant separation along with the 1^st^ component of the PCO. This is due to significantly higher concentrations of acetate, propionate, butyrate, adipate, pimelate, valerate, acetone, isobutyrate, and imidazole in RUM samples (Figure 6A) compared to DUO with significantly higher concentrations of, e.g., dimethylamine, cadaverine, trimethylamine, and a range of free amino acids (Wilcoxon test, all *p* ≤ 0.0005, Supplementary Table 4). The separation of RUM and DUO metabolomes was also seen when excluding the dominating SCFA, acetate, propionate, and butyrate (Figure 6B). Ruminal SCFA (Supplementary Figure 4), lactate (Supplementary Figure 5), and ketone bodies (Supplementary Figure 6) increased in both matrixes with increasing dietary concentrate level. RUM butyrate significantly increased during LPS challenge (+100 vs. 24hL, *p* = 0.04), which was confirmed by parallel GC–MS measurements of the samples (Supplementary Figure 7). RUM propionate and DUO acetate, propionate, butyrate, lactate, and isobutyrate significantly decreased during LPS challenge (*p ≤* 0.05).

Carnitine concentrations were significantly higher in DUO than RUM (*p ≤* 0.05) and not significantly different between DUO samples of control (CON) and carnitine-supplemented (CAR) animals (Supplementary Figure 8). There was no significant difference in the metabolite patterns between CON and CAR in both matrixes (Supplementary Figure 2C,D).

RUM alanine, glutamate, glycine, isoleucine, and leucine significantly increased after calving and feed change (−42 vs. +14) and decreased during LPS challenge significantly (+100 vs. 12hL, both *p* ≤ 0.05). In DUO at −42, 12hL, +118, and + 126, aspartate, proline, and histidine strongly increased, compared to all other time points (Supplementary Figure 9). DUO concentrations of formate showed a significant, four-fold increase from −14 to +100 and a significant decrease during the LPS challenge (+100 vs. 24hL, *p* ≤ 0.006). Free RUM glucose increased significantly after calving and/or feed change (−14 to +14) and decreased during LPS challenge (+100 vs. 12hL, both *p ≤* 0.02, Supplementary Figure 6). Alcohols including methanol, ethanol, and acetone significantly increased in RUM from *ap* to *pp* time points (*p ≤* 0.05) and decreased during LPS challenge (+100 vs. 12hL, *p ≤* 0.05; Supplementary Figure 10).

### Correlations between bacteriome and metabolome

Most metabolites in both matrixes correlated negatively with high α-diversities, such as in RUM samples valerate (*p* < 0.0001), trimethylamine, and propionate (*p* < 0.0001) as well as pimelate, alanine, and acetone (*p* < 0.0001). In DUO, glycine (*p* = 0.0004), isovalerate (*p* = 0.001), carnitine (*p* = 0.01), and isobutyrate (*p* = 0.006) negatively correlated with high microbial diversities. In rumen, 3-phenylpropionate correlated positively with high α-diversities (*p* = 0.02).

Coriobacteriaceae had particularly significant positive correlations among others with RUM valerate, pimelate and 4-hydroxybutyrate (all *p* < 0.0001) as well as with DUO glycine, propionate, and isobutyrate concentrations (Supplementary Table 5).

**Figure 7:**
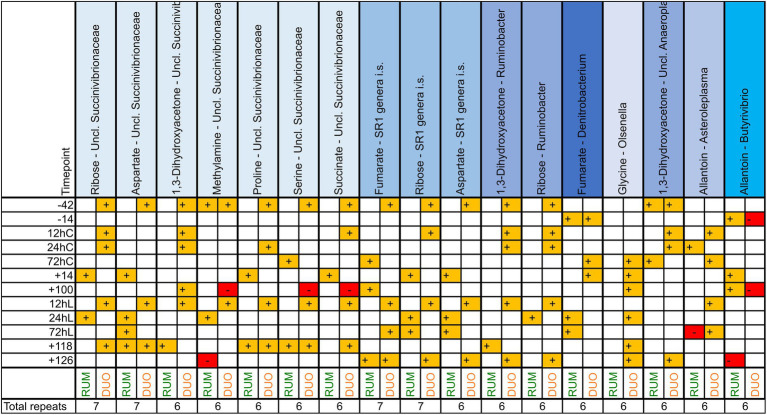
Significant (*p* ≤ 0.05) correlation combinations of ruminal (RUM, *N* = 66) and duodenal (DUO, *N* = 67) metabolites (*N* = 60) and genera (*N* = 102) repeating at least 7 times across 12 time points using Pearson product–moment multivariate correlations. Time points including a “–” or “+” indicate days *antepartum* or *postpartum* and time points including “hC” or “hL” are samples taken at 12, 24, or 72 h after calving or LPS challenge, respectively.

Correlating a great number of metabolites and genera and two matrixes at 12 different time points brings up a wealth of significant correlating combinations. Counting significant combination repeats between genera and metabolites, such as “SuccinivibrioFormate,” across all time points resulted into insights of those combinations appearing the most. The higher the repeat number of the combination across all time points, the more meaningful this combination may be to the host and the ecosystem. Unclassified *Succinivibrionaceae* was the genus correlating the most commonly with different metabolites across time and both matrixes, counting 15 metabolites (such as proline, aspartate, or ribose; Supplementary Table 6, the most frequent in Figure 7). Members of the genus *SR1 genera incertae sedis* (i.s.) were the second most common across time; however, *Bacteroides* genus was found to correlate with all 60 metabolites analyzed at least once across the whole trial period. Choline, in turn, was the most common metabolite correlator for bacteria, 86 out of 102 genera correlated with this metabolite, whereby formate was the most frequently repeating correlator across all time points and both matrixes, mostly with *Fusobacterium*, *Megasphaera*, *Ruminobacter*, *SR1 genera i.s.*, unclassified *Succinivibrionaceae* and unclassified *Bacteroidia*. Together with unclassified *Succinivibrionaceae* and SR1 genera i.s., *Olsenella* correlated most frequently and positively with the lactate concentration in RUM and DUO. The most frequent metabolite:genus combinations in both sample types were Fumarate:SR1 genera i.s, Ribose:SR1 genera i.s, Aspartate:unclassified Succinivibrionaceae and Ribose:unclassified Succinivibrionaceae.

Significant correlations between L-carnitine and a broad set of bacteria were observed for RUM and DUO uniquely but also shared ones (Supplementary Table 6). *Olsenella* was positively and unclassified *Bacteroidales* negatively associated with L-carnitine in both matrixes. In RUM, *Roseburia* (*p* < 0.0001) and *Syntrophococcus* (*p* < 0.0005) were positively and unclassified Proteobacteria negatively (*p* < 0.03) correlating with L-carnitine. In DUO, unclassified *Eubacteriaceae* (*p* < 0.04) and unclassified *Succinivibrionaceae* (*p* < 0.004) were positively associated with L-carnitine. Choline, carnitine, and trimethylamine are known to be involved in trimethylamine-N-oxide (TMAO) formation and at various time points correlating significantly with *Olsenella*, *Pseudoscardovia*, and unclassified *Veillonellaceae* abundances (Supplementary Table 7).

## Discussion

The present long-time study was conducted to identify the effect of L-carnitine and health challenges on the ruminal and duodenal microbiome at an animal individual level. Throughout 168 days, including calving and an LPS-induced inflammatory challenge, samples from eight fistulated dairy cows were collected. In addition to the broad phylogenetic analyses of the bacterial communities, 60 metabolites could be quantified in the RUM and DUO fluids. The animals’ observed life span included changes in the feed composition, reflected in changes in the microbiota and metabolite composition. Rumen and duodenal microbial community compositions showed very similar trends throughout the trial. This was also reported in culled lambs (Perea et al., 2017) but not in samples from culled dairy cows (Mao et al., 2015). The reasons for this high similarity could be the localization of the fistula, which was in front of the pancreatic duct, and this was possibly too close to the abomasum. For nutritional questions, the positioning of this fistula may be appropriate, yet 16S rRNA sequencing is according to the present work not sufficient in drawing the unique duodenal bacteriome, as not differentiating between live or dead cells.

In contrast to sequencing results, the metabolomes of both matrixes clearly clustered apart from each other, indicating a different mode of action of the bacterial communities and the host metabolism. This includes significantly higher abundances of fermentation products in the rumen (Deusch et al., 2017; Xue et al., 2020) and protein degradation-related metabolites (free amino acids, allantoin, and dimethylamine) in duodenal fluid samples. As the duodenal fistula was set before the pancreatic duct, it is assumed that especially the AA is greatly derived from bacteria. Concentration differences between RUM and DUO have to be discussed with care, as water, electrolytes, SCFA, and other metabolites are absorbed in the omasum, influencing duodenal metabolite concentrations. Thus, metabolic routes are hardly absolutely quantifiable.

### Rumen and duodenal unique characteristics blur due to high concentrate feeding

The introduction of a more nutrient-dense diet after calving resulted in a drop of pH values in RUM, also seen by Derakhshani et al. (2017), due to enhanced formation of SCFA and lactate. This may have caused unfavorable conditions for fibrolytic rumen bacteria supporting preceding studies (Saleem et al., 2012; Derakhshani et al., 2017; Plaizier et al., 2017). This feed change and/or the calving may have resulted into a displacement of the former microbiome by another, which is better adapted to higher digestive passage rates and lower pH levels. The free ruminal and duodenal AA increased, what may be due to an increase in microbial protein synthesis due to more energy in the diet. Also, this could mirror the higher feed intake and increase in dietary crude protein compared to *ap* time points, known for dairy cows (Derakhshani et al., 2017). An increased rumen wall epithelial abrasion (Hollmann et al., 2013) can also result in higher AA concentrations. Derakhshani et al. (2017) described that antepartal rumen microbiomes of individuals were more similar among each other compared to the postpartal phase, which could not be seen as clearly in the present work. Only before calving and feed change, RUM and DUO communities were significantly different from each other. To the best of our knowledge, this observation was not yet reported in the literature. It is not clear whether this merging in community structures of two physiologically different sites at early lactation was driven by the calving itself or the feed change. Since both events co-occurred simultaneously, subsequent effects cannot be assigned exclusively to one or the other cause. Although cellulolytic bacteria are known to be impaired at pH values below 6 (Lynd et al., 2002), *Fibrobacter* members were higher abundant in the acidified duodenal fluid. This may be due to the water absorption from ruminal digesta by the omasum and hereby the relative increase of fiber, to which *Fibrobacter* is commonly attached to (Hofmann, 1989).

### Bacterial taxa correlate with high or low diversities

The α-diversity along the timeline demonstrated that bacterial communities can be different between individuals, even though the feeding regimen and other environmental parameters were similar. The ruminal α-diversities increased toward calving and decreased thereafter, which was already found in other studies (Lima et al., 2015; Bach et al., 2019). Correlating the abundance of genera with the α-diversity values at same time points might uncover bacteria, which actively promote or benefit from a high or low microbial diversity. This was shown for *Olsenella*, which correlated negatively with high bacterial diversities and has been found to increase during high grain-induced subacute ruminal acidosis (Kim et al., 2018) as well as after calving (Bach et al., 2019). In the present study, significant positive correlations were observed in both matrixes between the Coriobacteriaceae, propionate, and a great number of free AA. It is known that this family forms a variety of aminopeptidases (Clavel et al., 2014), making AA available for other bacteria or the host, and has been associated with milk yield in past studies (Derakhshani et al., 2017). Right after LPS injection, ruminal *Olsenella* decreased (+100 vs. 12hL, Wilcoxon test *p* = 0.008), accompanied by reduced levels of its fermentation product lactate (Dewhirst et al., 2001).

### Uncovering an orchestrated bloom of *Bifidobacteria* after calving

Immediately after calving, Bifidobacterium’s abundance increased dramatically in both matrixes in five out of six animals sampled and disappeared again at +14. Interestingly, *Bifidobacterium* members decreased strongly with the number of lactation or age and were particularly low in RUM of animals that died in the course of the trial namely 039 and 248. The fecal microbiome analysis of these animals was done previously together with other non-fistulated cows. It could be shown that these animals belonged to a group of animals sorted in microbiome clusters, C-Spiro and C-Clos (Tröscher-Mußotter et al., 2021), which were characterized by high average days in illness, body condition scores (BCS), body weights (BW) as well as low milk protein and milk fat yields, fecal *Bifidobacterium* abundances, and residual energy intakes (REI) values around 0 or negative. In contrast, animal 306 was observed with considerably high *Bifidobacterium* abundances, and only had some skin issues, but no infectious diseases or claw infections. The fecal microbiome of this animal could be grouped to the third microbiome cluster (C-Bifi) of the previous study, which was characterized by medium milk yields, positive REI values, lowest BCS and body weights (BW) at high milk protein and milk fat yields (Tröscher-Mußotter et al., 2021). *Bifidobacterium* plays a significant role in the gut’s development and resilience as they can produce natural bacteriocins, SCFA, and conjugated linoleic acids (Arboleya et al., 2016). *Bifidobacterium* species were detected in fecal samples in cows before calving (Makino et al., 2013) and the vagina of pregnant cows (Laguardia-Nascimento et al., 2015). The transmission of bifidobacterial strains from the dam to calf was described *via* vaginal transmission during birth (Makino et al., 2013) and milk (Addis et al., 2016), and a colonization of *Bifidobacterium* within the calfs’ intestine during the first weeks of life was detected (Alipour et al., 2018). Our data for the first time showed a significant increase of *Bifidobacterium* spp. in rumen and duodenal fluid samples of cows 3 days *pp,* which has also been found in the fecal samples of the same animals (Tröscher-Mußotter et al., 2021). The calf was permitted to stay with the dam only until 1 day *pp*, allowing intense care, licking, and suckling. Hereby, it could be assumed, the cow inoculated herself orally with members of this bacterial genus. It can be hypothesized that this animal-collective increase of *Bifidobacterium* contributes to the controversially discussed entero-mammary pathway (Rainard, 2017), supporting the inoculation of *Bifidobacterium* members *via* milk. Hence, we suggest that the immediate separation of dam and calf after the newborns colostrum intake may be too early as data suggests a maternal initiation of probiotic stimuli for the calf at 3 days after calving. Unfortunately, the present study did not investigate the respective calves. The decreased abundance of *Bifidobacterium* species with aging is known for humans (Yatsunenko et al., 2012) and could be also seen in the present work. Yet, a bigger cohort is needed to verify this decline in dairy cows.

### The modern dairy cows’ dilemma

Cows in early lactation require high energy, which goes along with low feed intakes (Brandstetter et al., 2019). Energy density, therefore, has to be increased in the diet leading to an enhanced SCFA production in the rumen (Laffel, 1999; Saleem et al., 2012) and a decrease in rumen pH (*via* increase of SCFA and lactate; Yang and Beauchemin, 2006; Derakhshani et al., 2017). The high-energy demands cause body fat mobilization and lipid accumulation in the liver, with acetoacetate, 3-hydroxybutyrate (3Hbut), and acetone increasing. These are formed from acyl-CoA molecules from mitochondrial *β*-oxidation (Kuhla et al., 2016) and can be found in saliva, milk, and blood (Laffel, 1999) as well as, according to our study, in RUM and DUO samples. Together with free glucose, ketones positively correlated among each other in RUM and DUO (*r* ≥ 0.7) and increased in the rumen when concentration in the diet was increased. Interestingly animals with highest concentration of free ruminal glucose throughout the trial were those assorted to the animal cluster “C-Clos,” associated with longer days in illness, significantly higher milk somatic cell counts and blood mean platelet volumes at significantly lower dry matter intakes compared to the other two clusters within a related study including the same animals (Tröscher-Mußotter et al., 2021). If glucose is not available (Laffel, 1999), ketone bodies serve as an alternative energy source in peripheral tissue (Ruzsanyi and Kalapos, 2017). However, if excessively high, they can have neurotoxic properties (Fedorovich et al., 2018) and lead to ketosis and fatty liver development (Kuhla et al., 2016). Acetone increased significantly in the rumen fluid during early lactation, where it can be eructed through the esophagus or reduced to isopropanol by microbes (Bruss and Lopez, 2000). *Via* the hepatic portal route, isopropanol reaches the liver, where it is oxidized to acetone, which again is transferred in the aforementioned body fluids. This might explain the significant correlation between acetone and isopropanol in the present study. Emissions are promoted *via* breathing (Ruzsanyi and Kalapos, 2017) and rumination. However, as rumination is reduced with higher concentrate levels in the diet due to the reduction of physical effective fiber (Brandstetter et al., 2019), an accumulation of acetone and other alcohols in rumen could be measured, possibly affecting microbial membranes in phospholipid content, fluidity, and stress the former microbes. Yet, this depends on the general membrane structure and may target individual groups of bacteria more than others (Dyrda et al., 2019). DUO samples had significantly higher 3Hbut concentrations; other ketone bodies were lower than in RUM samples. Intestinal 3Hbut was lately reported as an important driver for intestinal cell differentiation and gut homeostasis (Wang et al., 2017). Together, the aforementioned processes might lead to a vicious cycle during the first 2 weeks of lactation and cause higher incidences of laminitis, ketosis, abomasal displacements, and milk fever (Goff and Horst, 1997), which can be confirmed by our findings examining animals with increased health issues, including laminitis, skin erosions and udder injuries.

### The microbial “airbag” hypothesis

After the LPS injection, cows stopped the intake of feed and showed clinical symptoms, including fever, diarrhea, muscle twitching, sweating, and a shutdown of rumination and rumen activity for several hours (Meyer et al., 2021). Calving is often preceded by a feed refusal and decreased rumination activity. It is assumed that simpler carbohydrates in the rumen during both challenges were quickly degraded by the microbiome, whereas fibers and coarse grain particles were not further mechanically crushed as rumination stopped and gut motility was reduced (Meyer et al., 2021). This resulted in an increase of fibrolytic bacteria such as *Ruminococcus*, unclassified *Ruminococcaceae*, and *Butyrivibrio* during both challenges, as they benefited from the increased retention time and best adapted to this substrate. Similar conclusions were drawn in steers during fasting (Kim et al., 2019). Ruminal glucose, propionate, lactate, ketones, and alcohols significantly declined, which are metabolites related to high milk production and high grain feeding (Saleem et al., 2012; Derakhshani et al., 2017) but also to fatty liver disease and ketosis (Mahapatra and Sahoo, 2006). In addition, ruminal SCFA remained unaffected and butyrate even increased, respectively, during the LPS challenge. Butyrate increased epithelial thickness and enlarging surface area of ruminal epithelium in goat studies (Malhi et al., 2013). It therefore could be suggested that butyrate strengthened the cows intestinal conditions during this acute stress phase. It is suggested that the aforementioned LPS illness symptoms were mainly affecting the bacterial composition *via* fasting as described in other studies working with cows in stress and non-intestinal diseases, induced by transportation (Deng et al., 2017), ketosis (Wang et al., 2012) or heat (Zhao et al., 2019). Nevertheless, this bacterial community process was never described as a possible strategy to overcome critical phases.

For some taxa, the LPS challenge had a significantly stimulating effect, such as for duodenal unclassified *Clostridiales* and the rumen Proteobacteria population. Beyond the days of the LPS challenge, ruminal unclassified *Gammaproteobacteria* were significantly promoted by this induced inflammation, whereas duodenal *Veillonellaceae* were significantly decreased. Similar results were observed in human patients with increased stress and irritated bowel disease (Humbel et al., 2019). Also, increased ruminal abundances of Proteobacteria have been associated with dysbiosis in ruminants (Auffret et al., 2017) and in general with the postpartal, high-concentrate diet period (Derakhshani et al., 2017). Hence, we assume, that dietary fiber and fibrolytic bacteria buffered the severity of the LPS challenge, thus acting as a microbial airbag, by keeping up energy supply (SCFA), reducing beta oxidation and hereby reducing ketone body formation, stabilizing gut integrity *via* butyrate production. Even though health-promoting bacteria, aided the animals in the acute phase of the LPS challenge *via* fiber fermentation, taxa of potentially pathogenic bacteria like unclassified *Gammaproteobacteria* may have had a benefit from this induced infect in the long run.

### Fates of L-carnitine, choline and formate in the ruminant ecosystem

In the present study, L-carnitine was supplemented in the feed of four animals to enhance the efficiency in the fatty acid transport into the mitochondria and thus, to likely support the overall physiological capacity of the cows (Meyer et al., 2020). L-carnitine serves as a nutrient source for bacteria and enhances their robustness in various environments and also can be synthesized by anaerobic bacteria (Meadows and Wargo, 2015). In the present study, the supplementation did not affect the rumen or duodenal total bacterial community composition nor the metabolome. As a rumen-protected L-carnitine product was applied, significant ruminal concentration differences between CON and CAR animals were not found, and duodenal concentrations were higher compared to rumen fluid samples. High amounts of intestinal L-carnitine and choline can increase free trimethylamine (TMA) in the animal, which is further oxidized in the liver to TMAO (Roncal et al., 2019). Genera such as *Olsenella* in both matrixes, *Pseudoscardovia,* and unclassified *Veillonellaceae* in RUM and *Campylobacter* in DUO may be involved in converting L-carnitine and choline to trimethylamine, as they were significantly correlating with the above-mentioned metabolites in the present study. L-carnitine and choline, irrespective of the supplemented group, highly correlate with TMA in RUM but not in DUO (Supplementary Figure 11), which is why we assume that this conversion rather takes place in the rumen than in the duodenum.

Carnitine was not significantly increased in DUO samples of L-carnitine-supplemented animals. There is a lack of comparable concentration data for duodenal fluid samples but the present NMR results of rumen fluid samples are in line with Saleem et al. (2013) (comparing time point −42). RUM and DUO laboratory protocols only differed in types extraction buffers, hereby, we suggest that the quality of analysis was similar. As the CAR supplementation resulted in significantly higher blood L-carnitine concentrations in CAR animals (Meyer et al., 2020, 2021), we can confirm that at some point the rumen-protected L-carnitine was absorbed from the intestine. It therefore could be hypothesized that L-carnitine was already metabolized up to the duodenal fistula.

A metabolite interacting with a large number of bacterial genera was choline, which is known to provide an array of health-, reproduction-, and production-related functions in the ruminant (Jayaprakash et al., 2016). For example, it improves lipid transport in the blood and NEFA decrease in the liver. It is mostly ingested *via* feed in the form of lecithin, which is hydrolyzed to choline by intestinal mucosal cells and pancreatic enzymes (Pour et al., 2014). Also, choline can be endogenously synthesized and is rapidly degraded in the rumen (Pinotti et al., 2020). However, the present data confirmed that there were significantly (*p* < 0.05) higher choline concentrations found in DUO, either deriving from the microbial activity or a host-derived synthesis of choline in between the rumen and duodenum, as no rumen-protected choline was fed (Meyer et al., 2020). Formate is a product of polysaccharide fermentation, serves as H_2_ sink and, as also observed in the present work, increases with increasing concentrate in diet (Martinez-Fernandez et al., 2016). As the rumen pH with inclusion of nutrient-dense diets decreases, methanogenesis is inhibited. The resulting accumulation of formate (Martinez-Fernandez et al., 2016) may disturb microbial metabolisms and growth (Kelly et al., 2022). As fermentation is the greatest purpose of the rumen, with formate and H_2_ as end products, it is not surprising that in the present work, formate was found to be the most prominent correlator for bacteria. These findings underline the importance of ruminal formate metabolism and its role for rumen microbial syntrophy. Among others, ribose was often involved in correlation combinations with genera in both matrixes and challenges. This may be due to low energy ingestion that led to a degradation of microbes and therefrom a degradation of nucleic acids hereby releasing ribose, which then serves as substrate for other bacteria.

### Conclusion

The present long-term study showed for the first time how the bacteriome of cows’ cope with the challenge of transition into lactation and an LPS injection using a multi-omics approach. Rumen and duodenal fluid bacteriomes were significantly different before calving but thereafter, very similar along a 126-day period. *Bifidobacterium* bloomed up at 3 days after calving, which was only described for the calves’ microbiome before, opening new hypotheses of why this is happening in the dam. The LPS challenge led to a severe illness; nevertheless, data suggests that increasing, health-promoting bacteria cushion the severity of the disease during the acute phase, thereby acting as a microbial “airbag.” Duodenal fluid samples were, to the best of our knowledge, not yet characterized using NMR analysis, which exposed microbial similitude, not automatically implying functional equivalence. A new data analysis approach revealed choline as the most correlative metabolite and formate as the most iterative correlating metabolite with rumen and duodenal genera. Further research is needed, perhaps with appropriate cows on pasture all year round and thus on a largely stable diet, even during the transition period, in order to understand the basic biology behind a cow in lactation and its microbiome.

## Materials and methods

### Ethical statement

The experiment was carried out at the experimental station of the Institute of Animal Nutrition, Friedrich-Loeffler-Institute (FLI), in Braunschweig, Germany in accordance with the German Animal Welfare Act approved by the LAVES (Lower Saxony Office for Consumer Protection and Food Safety, Oldenburg, Germany) (AZ33.19-42502-04-16/2378).

### Animal experiment and sampling

This study is part of the cooperative project “Mitochondrial functionality in dairy cows” (MitoCow), funded by the German Research Foundation (DFG) including 59 multiparous German Holstein-Friesian dairy cows (Meyer et al., 2020, 2021). Within this cohort, eight cows were equipped with fistulae in rumen and duodenum. These cows were investigated in the present study. The eight animals were randomly separated into a carnitine (CAR) and control group (CON; Figure 1). CAR animals received 125 g of Carneon 20 Rumin-Pro (Kaesler Nutrition GmbH, Cuxhaven, Germany), which is equivalent to 25 g of rumen-protected L-carnitine per cow and day included in concentrate feed. This amount is not suggested to result into depression or stimulation of feed intake (Meyer et al., 2020). CON animals received an equivalent amount of fat (BergaFat F-100 HP, Berg+Schmidt GmbH & Co. KG, Hamburg, Germany) compared to CAR animals, to compensate the Carneon supplementation. During the dry period, silage and hay were offered to the animals with additional access to pasture. Two weeks before initiating the experiment, cows were fed a complete ration for dry cows similar in composition to the control ration fed from day 42 *antepartum* (*ap,* −42). This consisted of 80% roughage and 20% concentrate until calving (day 0) and contained the supplements in the concentrate feed. From the day of calving up to day 14 *postpartum* (*pp*, +14), the amount of concentrate was gradually increased up to a ratio of 50:50. This regimen was continued until the end of the trial. Roughage comprised 70% maize silage and 30% grass silage; water was offered *ad libitum*. Details about the animal experimental setup and feeding are published for the calving period (days −42 to +110; Meyer et al., 2020) and the LPS challenge (days +110 to +126; Meyer et al., 2021). In short, LPS challenge was conducted at day +111, whereby 0.5 μg/kg BW LPS (E. coli, Serotyp O111:B4, Sigma Aldrich, L2630, St. Louis, Missouri, USA) per cow were injected into the *Vena jugularis externa*. There were no negative controls, all animals were challenged.

Rumen (RUM) and duodenum (DUO) samples were taken regularly at 7 am after milking and at 12 time points between day −42 and day +126, including the sampling time points 12, 24, and 72 h after calving (12hC, 24hC, 72hC) and an LPS-induced inflammatory challenge at day +111 (12hL, 24hL, 72hL; Figure 1), where the animals are supposed to be out of negative energy balance (Butler, 2005). RUM samples were collected from the dorsal rumen sack *via* fistula (inner diameter: 10 cm), using a manual pump, which was thoroughly washed with water between samplings and animals, and fitted with a sieve that filtered larger feed particles. The first spills of pumped rumen fluid were discarded. Duodenal fistulae (inner diameter: 2 cm), placed before the pancreatic and bile duct, were equipped with simple screwing caps. After opening them, the first spill of duodenal fluid was discarded and the further fluid was collected. In total, 73 rumen fluid and 75 duodenal fluid samples with each 30 ml per time point were collected, pH was measured right after sampling (model pH 525; WTW, Weilheim, Germany), and samples were immediately stored at −80°C until further processing. During the trial, animal 039 was euthanized right after time point +14 due to a multifactorial inflammation, and animal 248 died shortly after the samples at time point 12hL were taken after an inflammatory shock. However, samples deriving from these animals have been included in this study.

### DNA extraction and Illumina amplicon sequencing

DNA was mechanically extracted from 350 μl sample material following the FastDNA™ Spin Kit (MP Biomedicals, Solon, OH, USA) for soil protocol with minimal changes as described previously (Burbach et al., 2016). DNA quantity and quality were measured using NanoDrop ONE (Thermo Fisher Scientific, Darmstadt, Germany). The V1-2 region of the 16S rRNA gene was targeted to construct an amplicon Illumina sequencing library using a three-step PCR amplification approach. The first PCR was used to increase the number of amplicons using Takara PrimeSTAR® HS DNA Polymerase (Takara Bio USA Inc.) and bacterial primers according to Kaewaptee et al. (Kaewtapee et al., 2017) applying a denaturation step for 3 min at 95°C. This was followed by a 10 x cycling program of denaturation at 98°C for 10 s, primer annealing at 55°C for 10 s and extension at 72°C for 45 s with a final extension at 72°C for 2 min. The 2^nd^ and 3^rd^ PCR were conducted to attach barcodes and indexes to the amplicons (Kaewtapee et al., 2017). PCR amplicons were verified by agarose gel electrophoresis and were normalized using Sequalprep™ Normalization Kit (Thermo Fisher Scientific), following the producers’ manual. Samples were pooled per index and purified with MinElute PCR Purification Kit (Qiagen, Hilden, Germany) and the final DNA concentration was measured using Qubit® 2.0 fluorometer (Invitrogen, Waltham, Massachusetts, USA). Samples were sequenced with 250 base pairs (bp) paired-end sequencing chemistry applied on an Illumina MiSeq platform.

### Sequencing data analysis

Raw sequence reads obtained from Illumina MiSeq system (Illumina, Inc., San Diego, CA, USA) were analyzed using QIIME2 v 2019^1^ (Bolyen et al., 2019). Demultiplexing, quality filtering, and trimming of sequencing reads were done using the default parameters of the pipeline with a maximum sequence length of 360 bp. The resulting dataset went through steps of denoising, dereplication, chimera removal, and merging through DADA2 (Callahan et al., 2016). Taxonomy assignation was done with a pre-fitted sklearn-based classifier (Pedregosa et al., 2011), using the SILVA Database (Release 132)^2^ (Quast et al., 2012). A total of 3,270,150 reads were clustered into 19,409 OTUs at 97% identity, further filtered by cutting all OTUs related to chloroplasts (3 OTUs). Additionally, those OTUs with 1 to 10 reads in total across all rumen and duodenal fluid samples and less than or equal to 100 reads per sample were discarded, resulting in 2,658 OTUs and 19,751 ± 1,039 average reads per sample. Six samples with less than 4,000 reads were deleted, resulting in 68 RUM and 75 DUO sequenced samples. The closest representative of each OTU sequence was manually identified using the classifier tool of the Ribosomal Database Project (Wang et al., 2007; Cole et al., 2014; version 2.11) and the taxonomy levels were screened and adjusted as described by Yarza et al. (Yarza et al., 2014).

### Nuclear magnetic resonance measurement

A total of 71 RUM and 67 DUO samples were analyzed using the 600 MHz-Bruker AVANCE III HD nuclear magnetic resonance spectroscope as described previously (Deusch et al., 2017). Sterile-filtered and vacuum-dried RUM samples were reconstituted using 50 mM sodium phosphate buffer at pH 7, and DUO samples with citrate–phosphate buffer at pH 5, both in deuterium oxide (D_2_O, 99.9% Sigma-Aldrich, Germany) containing 5 mM 3-(trimethylsilyl)propionic-2,2,3,3-d_4_ acid sodium salt (Sigma-Aldrich, Germany). Data were processed using Chenomx NMR Suite 8.2 software (Weljie et al., 2006; Inc., Edmonton, AB, Canada) to identify and quantify 60 metabolites including, among others, fatty, amino and carboxylic acids, amines, and carbohydrates. At a pH of 5, a reliable identification and subsequent quantification of urea and many other compounds is not possible. This accounts as well to other buffers at a pH of 5 and is not attributed to citrate–phosphate buffer exclusively. NMR data analysis was done as described by Deusch et al. (2017).

### Statistical analysis

PRIMER-E (Plymouth Marine Laboratory, UK; Clarke et al., 2014) was used for statistical and graphical analysis and the calculation of the Shannon diversity index (α-diversity). The Bray–Curtis coefficient (Bray and Curtis, 1957) was used to create similarity matrixes and principal coordinates analysis (PCO) plots. Global-R and *p*-values were generated using one-way analysis of similarity (ANOSIM) and permutational multivariate analysis of variance (PERMANOVA). This approach was used to evaluate similarity between different groups (e.g., CON vs. CAR, time points). The similarity percentages (SIMPER) tool was applied to find the main contributors of differences between groups. In JMP® Pro 15.2.1, Shapiro test was used to test the normality of distribution of bacteria and metabolites and Wilcoxon/Kruskal–Wallis test was used to test for significance. Barplots and boxplots were drawn using JMP® Pro 15.2.1 software. The same software was used for the Pearson’s product–moment correlation coefficient measures between metabolites and genera.

## Data availability statement

Sequences were submitted to European Nucleotide Archive (https://www.ebi.ac.uk/ena), accession number PRJEB41061. NMR data are available from the corresponding author upon request.

## Ethics statement

The animal study was reviewed and approved by Lower Saxony Office for Consumer Protection and Food Safety, Oldenburg, Germany. Written informed consent was obtained from the owners for the participation of their animals in this study.

## Author contributions

KH, JF, SvD, and JS: conceptualization. KH, JF, SvD, and JS: project administration and funding acquisition. KH, JF, SvD, AC-S, and JS: supervision. JT-M and JS: writing original draft. JT-M, SiD, AC-S, and JS: methodology. JT-M, DB-M, SiD, and AC-S: formal analysis and software. JT-M, AC-S, and JS: investigation and visualization. JT-M, SiD, DB-M, JF, SvD, AC-S, KH, JS: review and editing. All authors contributed to the article and approved the submitted version.

## Funding

We are thankful to the DFG for funding (202989534). The authors acknowledge support by the High Performance and Cloud Computing Group at the Zentrum für Datenverarbeitung of the University of Tübingen, the state of Baden-Württemberg through bwHPC and the German Research Foundation (DFG) through grant no INST 37/935-1 FUGG.

## Conflict of interest

The authors declare that the research was conducted in the absence of any commercial or financial relationships that could be construed as a potential conflict of interest.

## Publisher’s note

All claims expressed in this article are solely those of the authors and do not necessarily represent those of their affiliated organizations, or those of the publisher, the editors and the reviewers. Any product that may be evaluated in this article, or claim that may be made by its manufacturer, is not guaranteed or endorsed by the publisher.

## Footnotes

^1^ http://qiime2.org/

^2^ https://www.arb-silva.de/
