# Supplementary material for: Cow’s microbiome from antepartum to postpartum: A long-term study covering two physiological challenges
Source: Front Microbiol. 2022 Nov 16;13:1000750. doi: 10.3389/fmicb.2022.1000750 (PMC9709127; doi:10.3389/fmicb.2022.1000750)
Supplement: Supplementary file 7 [file Data_Sheet_2.PDF]

Supplementary Material

Cow's microbiome from antepartum to postpartum: a long-term study covering two physiological challenges

Johanna Tröscher-Mußotter[1,2], Simon Deusch[2], Daniel Borda-Molina[2], Jana Frahm[3], Sven Dänicke[3], Amélia Camarinha-Silva[1,2], Korinna Huber[1,2], Jana Seifert[1,2*]

[1] HoLMiR - Hohenheim Center for Livestock Microbiome Research, University of Hohenheim, Leonore-Blosser-Reisen-Weg 3, 70599 Stuttgart, Germany

[2] Institute of Animal Science, University of Hohenheim, Emil-Wolff-Str. 6-10, 70599 Stuttgart, Germany

[3] Institute of Animal Nutrition, Friedrich-Loeffler-Institut, Federal Research Institute for Animal Health, Bundesallee 37, 38116 Braunschweig, Germany

*** Correspondence:**
Jana Seifert
jseifert@uni-hohenheim.de

1 Supplementary Data

Supplementary Data include Table 1 to Table 6 and are available online.

2 Supplementary Figures and Tables

2.1 Supplementary Figures

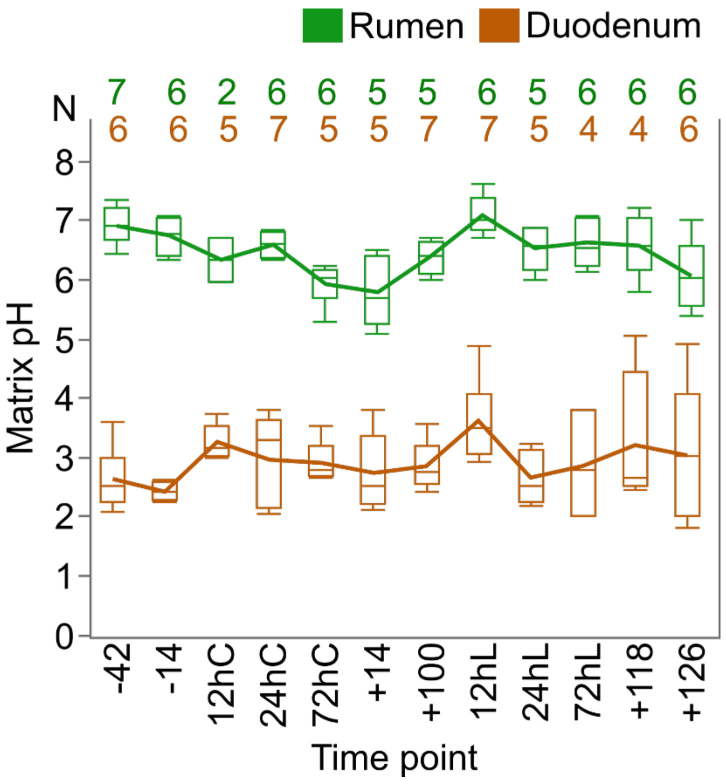

Rumen		
Time point	vs. Time point	*p*-Value
+14	24hC	0.049
+118	72hC	0.045
12hL	24hC	0.037
+14	-14	0.037
24hL	12hL	0.037
24hL	72hC	0.036
+100	-42	0.030
12hL	+100	0.027
72hL	72hC	0.023
72hC	-14	0.014
72hC	24hC	0.014
12hL	+14	0.012
+14	-42	0.009
12hL	72hC	0.008
72hC	-42	0.003
**Duodenum**		
Time point	vs. Time point	*p*-Value
12hL	-14	0.014
12hC	-14	0.020
72hC	-14	0.020
12hL	-42	0.020
12hL	72hC	0.036
12hL	+100	0.036

Supplementary Figure 1 Rumen (green, N=66) and duodenal (orange, N=67) pH values along the trial period. Trend lines connect means across the trial phase. N refers to sample number included per time point. Time points including a "–" or "+" indicate days *antepartum* or *postpartum* and time points including "hC" or "hL" are samples taken at 12, 24 or 72 hours after calving or LPS challenge, respectively. Table on the right includes significantly different time points based on non-parametric Wilcoxon test.

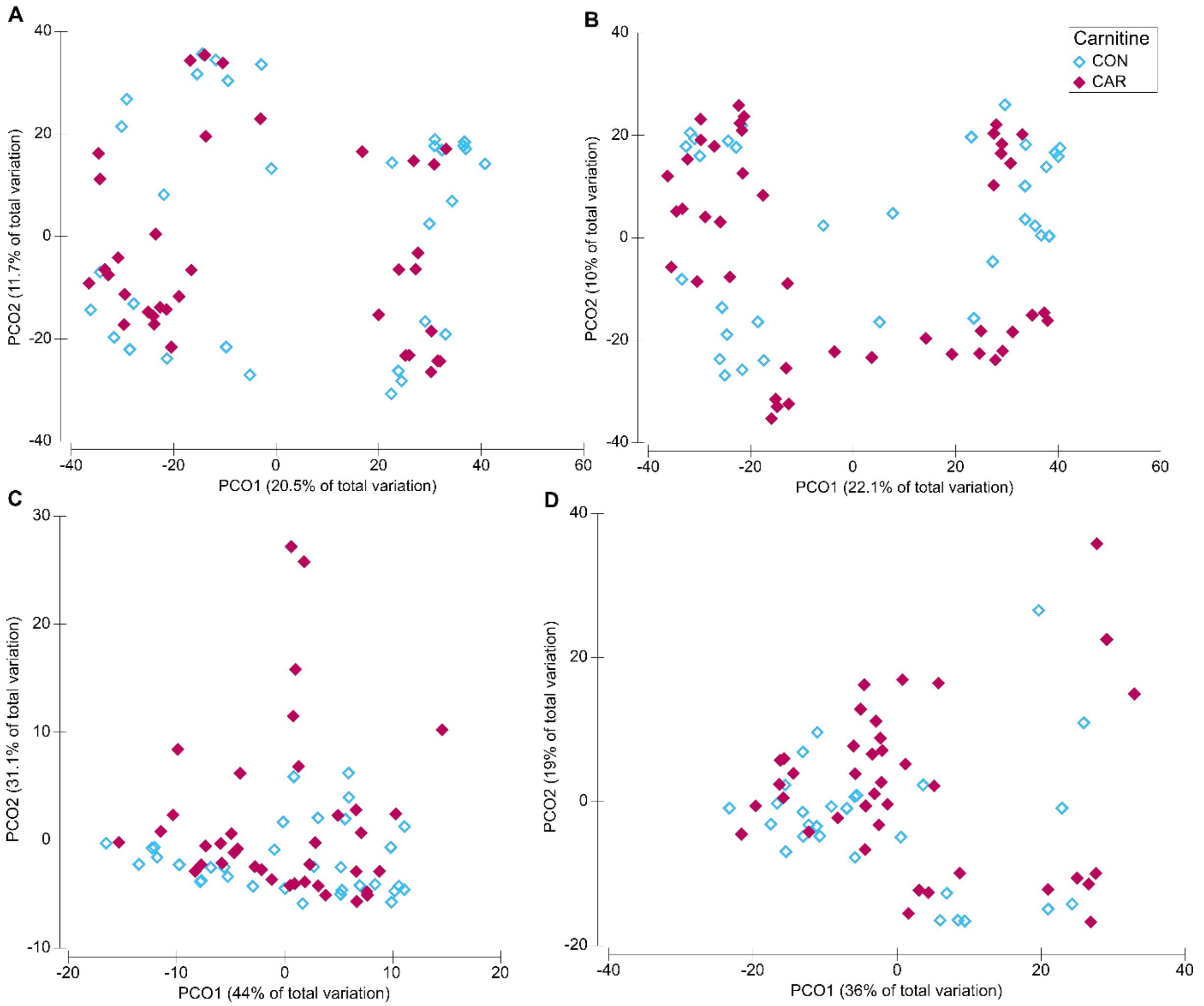

Supplementary Figure 2 PCO plot on bacterial community data at 12 points of **(A)** rumen (N=68, ANOSIM: Global-R=0.06, p=0.02) and **(B)** duodenal fluid samples (N=75, ANOSIM: Global-R =0.062, p=0.02) and metabolite data (60 metabolites) including SCFA **(C)** for rumen (N=71, ANOSIM: Global-R =-0.004, p=0.5) and **(D)** for duodenal fluid samples (N=67, ANOSIM: Global-R = - 0.003, p=0.47). Samples of the control group (CON) are depicted as blue/unfilled symbols and carnitine group (CAR) as red/filled symbols.

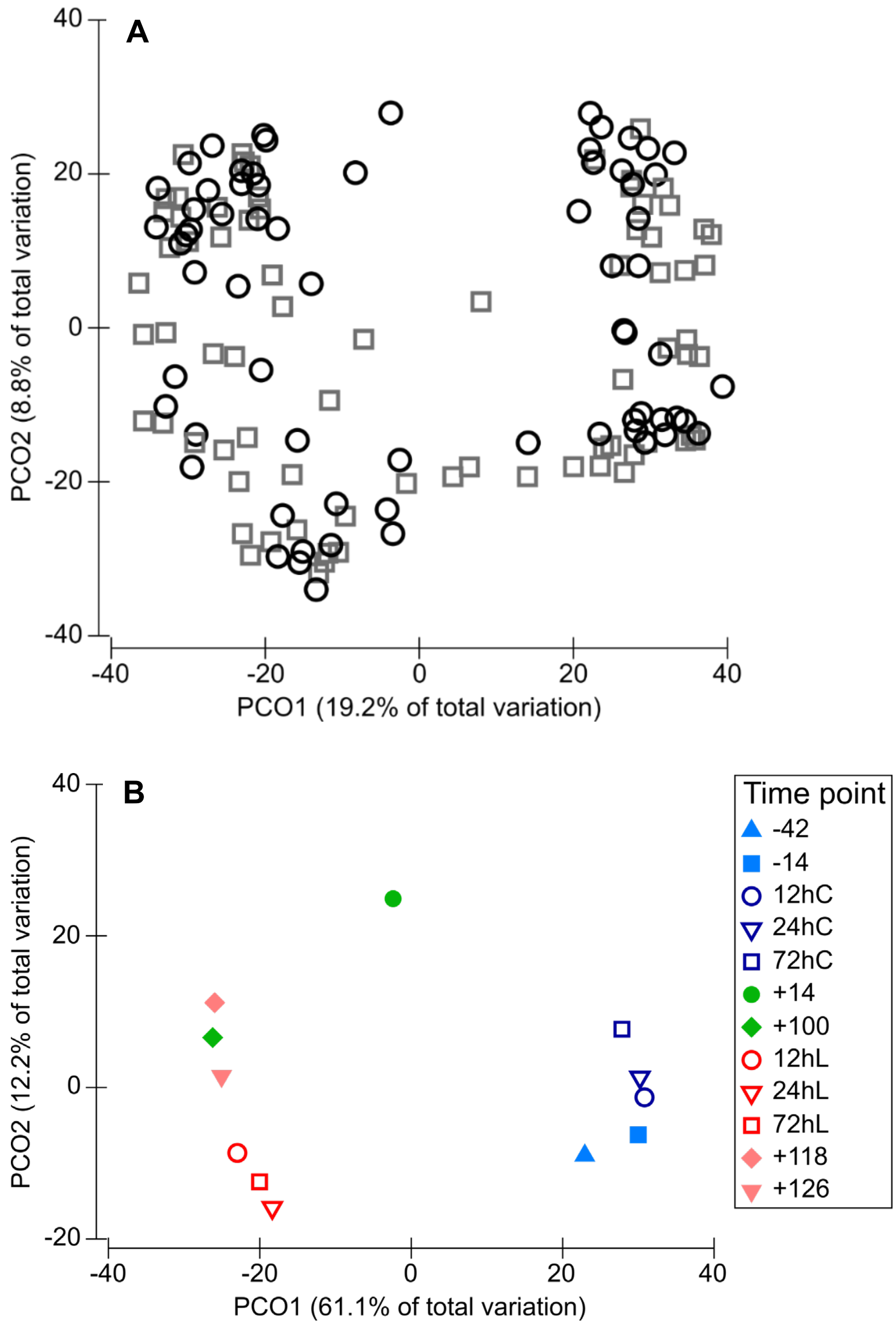

Supplementary Figure 3 (A) PCO plot on microbial communities of rumen (N=68, black circles) and duodenal fluid samples (N=75, grey rectangles) and **(B)** variation of the bacteriome along the full sampling period. This PCO plot on OTU level includes 143 samples grouped amongst 12 time points depicted as centroids. Matrix definitions are neglected. Time points including a "–" or "+" indicate days *antepartum* or *postpartum* and time points including "hC" or "hL" are samples taken at 12, 24 or 72 hours after calving or LPS challenge, respectively.

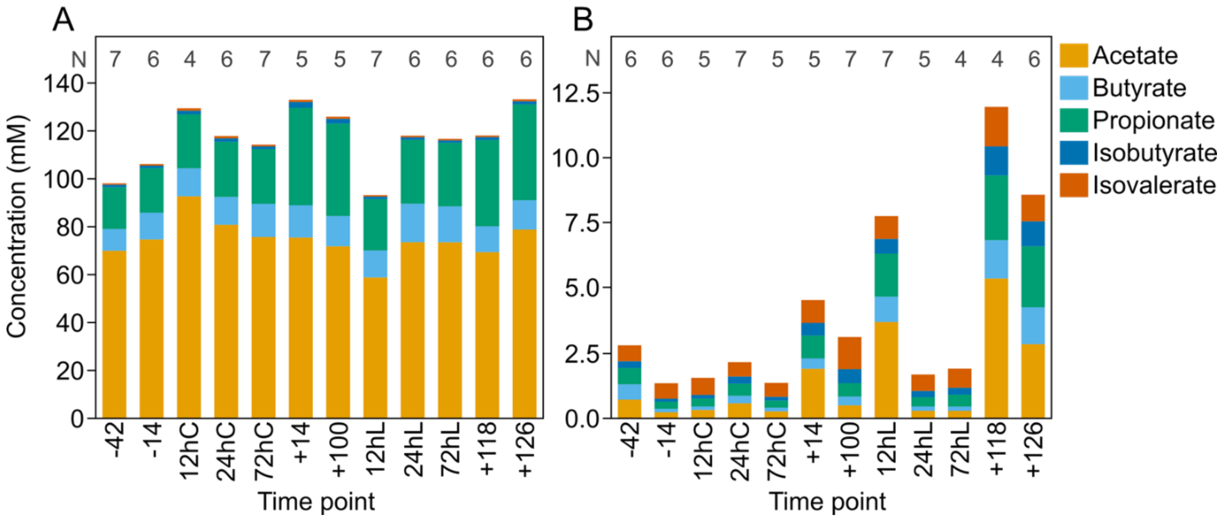

Supplementary Figure 4 Mean concentrations of SCFA in **(A)** rumen fluid and **(B)** duodenal fluid samples. N refers to sample number included per time point. Time points including a "–" or "+" indicate days antepartum or postpartum and time points including "hC" or "hL" are samples taken at 12, 24 or 72 hours after calving or LPS challenge, respectively.

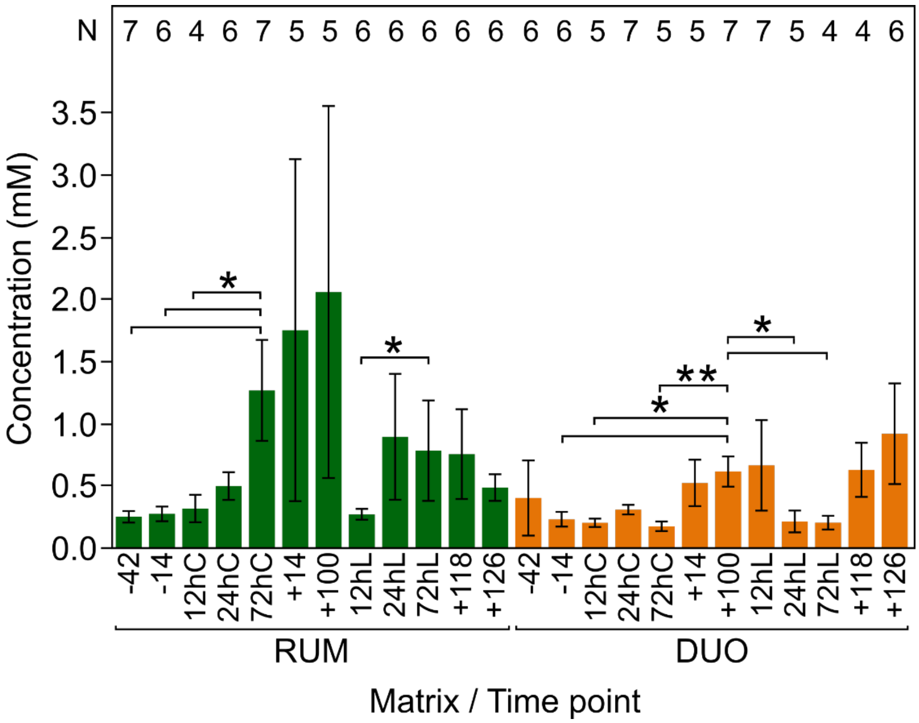

Supplementary Figure 5 Lactate concentration (mM) in rumen (RUM) and duodenal fluid samples (DUO) throughout the trial. N refers to sample number included per time point, error bars indicate SEM. Time points including a "–" or "+" indicate days *antepartum* or *postpartum* and time points including "hC" or "hL" are samples taken at 12, 24 or 72 hours after calving or LPS challenge, respectively. Asterisk indicates non-parametric Wilcoxon test, whereby *$p \leq 0.05$ and **$p \leq 0.009$.

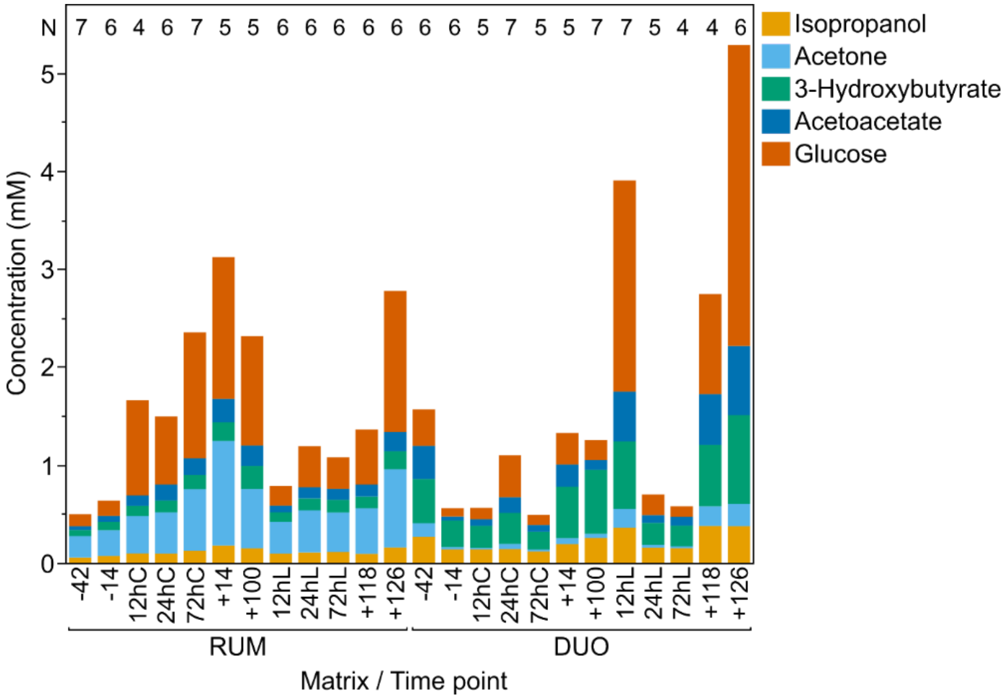

Supplementary Figure 6 Metabolites formed in beta-oxidation processes and glucose concentration in rumen (RUM) and duodenal fluid samples (DUO). Time points including a "–" or "+" indicate days *antepartum* or *postpartum* and time points including "hC" or "hL" are samples taken at 12, 24 or 72 hours after calving or LPS challenge, respectively.

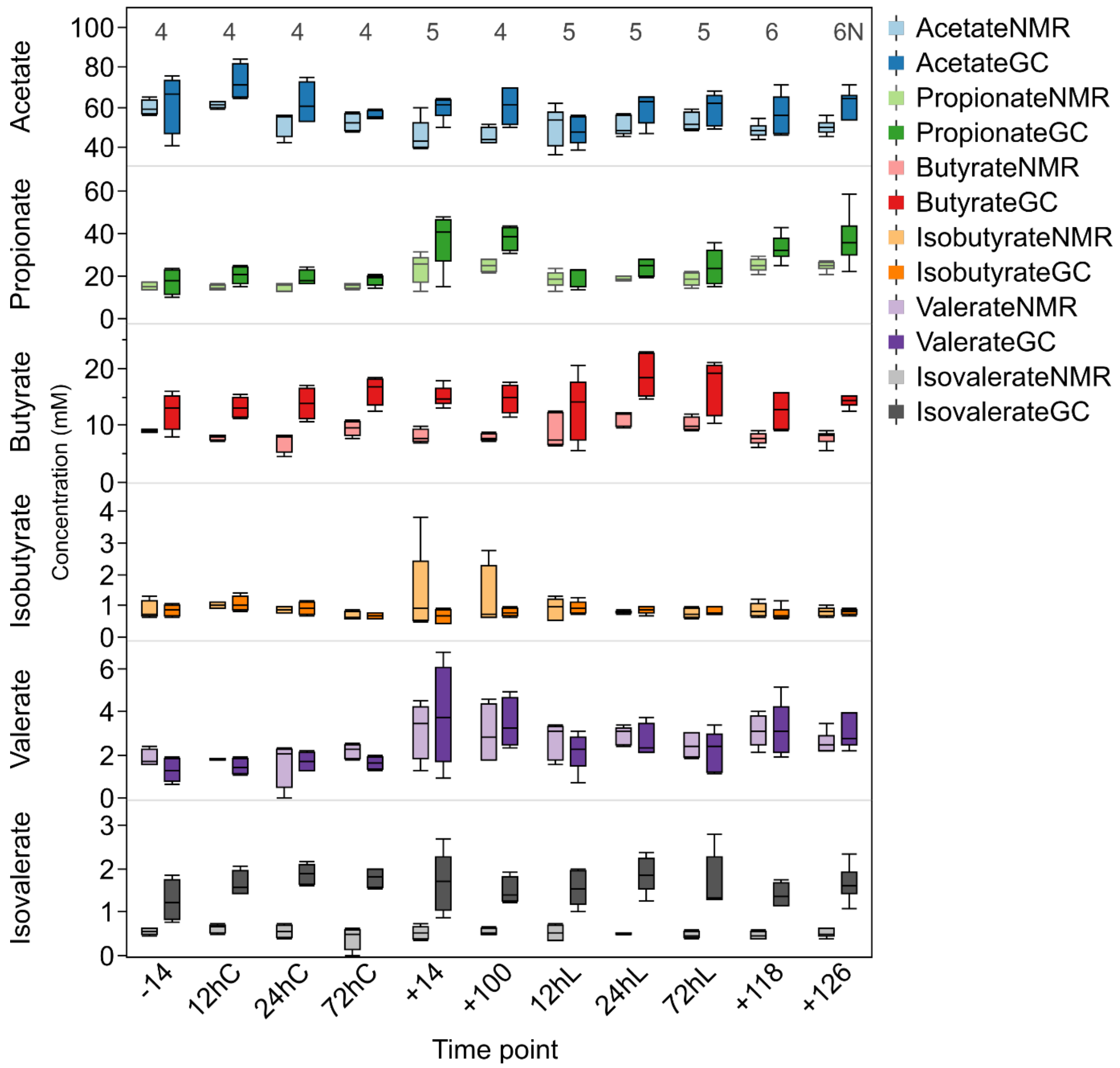

Supplementary Figure 7 Rumen samples at 11 time points analyzed for short chain fatty acids (SCFA; acetate, propionate, butyrate, valerate, isovalerate and isobutyrate) in mM using nuclear magnet resonance (NMR) analysis (see Materials and Methods) and a gas chromatography (GC) approach as described in Geissler, C., Hoffmann, M. & Hiokel, B. Ein Beitrag zur gaschromatographischen Bestimmung flüchtiger Fettsäuren. Arch. f.ür. Tierernaehrung 26, 123–129 (1976)). N refers to sample number included per time point, error bars indicate SEM. Time points including a ‚–' or ‚+' indicate days antepartum or postpartum and time points including ‚hC' or ‚hL' are samples taken at 12, 24 or 72 hours after calving or LPS challenge, respectively.

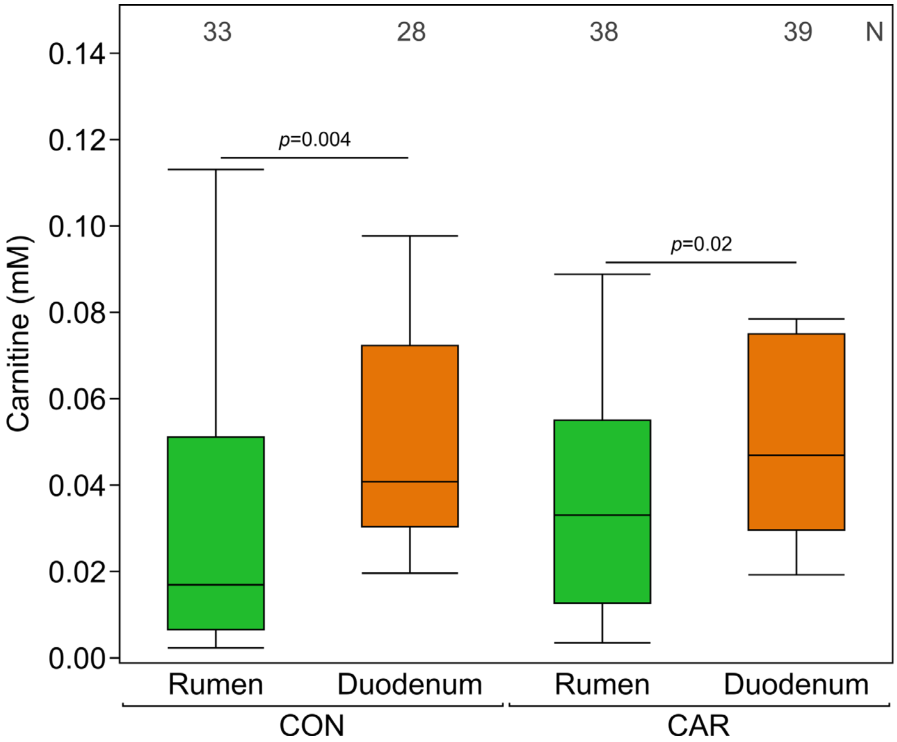

Supplementary Figure 8 Carnitine concentration (mM) in rumen (green, N=71) and duodenum (orange, N=67) samples of control (CON, N=61) and carnitine supplemented (CAR, N=77) animals throughout all time points and across all animals. N refers to sample number included per matrix. P-values refer to non-parametric Wilcoxon test.

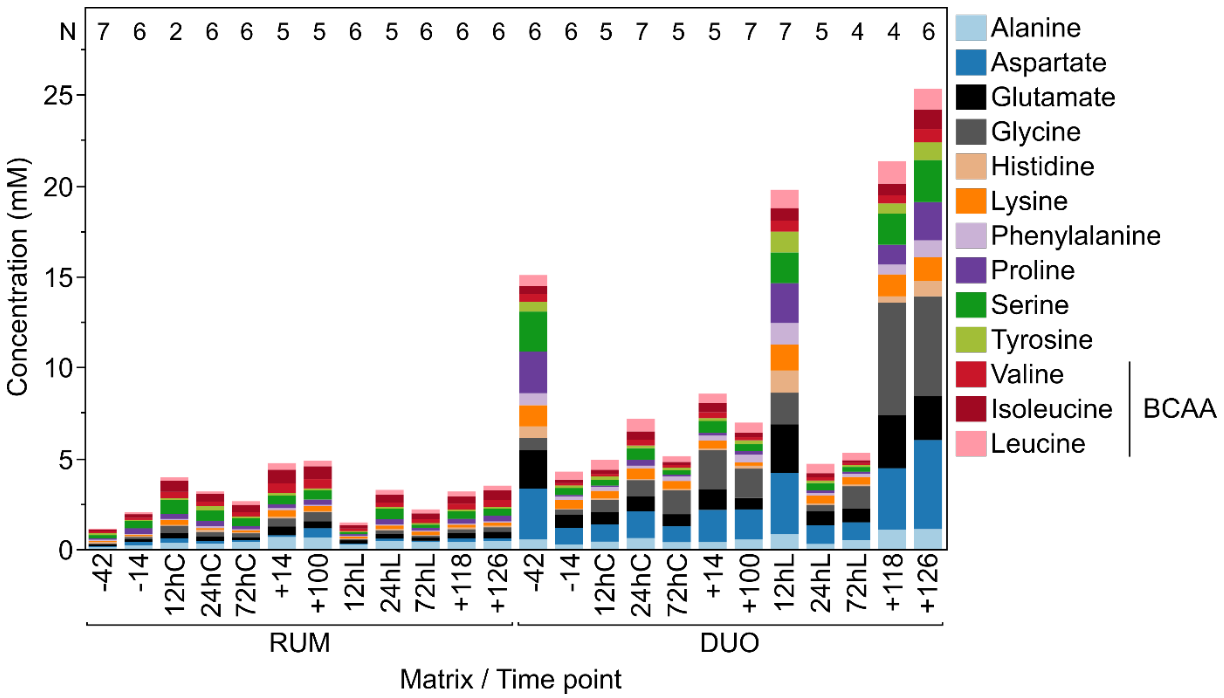

Supplementary Figure 9 Concentration (mM) of amino acids and branched chain amino acids (BCAA, in red shades) measured by NMR spectroscopy across the complete trial phase in rumen (RUM) and duodenum (DUO) samples. N refers to sample number included per time point. Time points including a "–" or "+" indicate days antepartum or postpartum and time points including "hC" or "hL" are samples taken at 12, 24 or 72 hours after calving or LPS challenge, respectively.

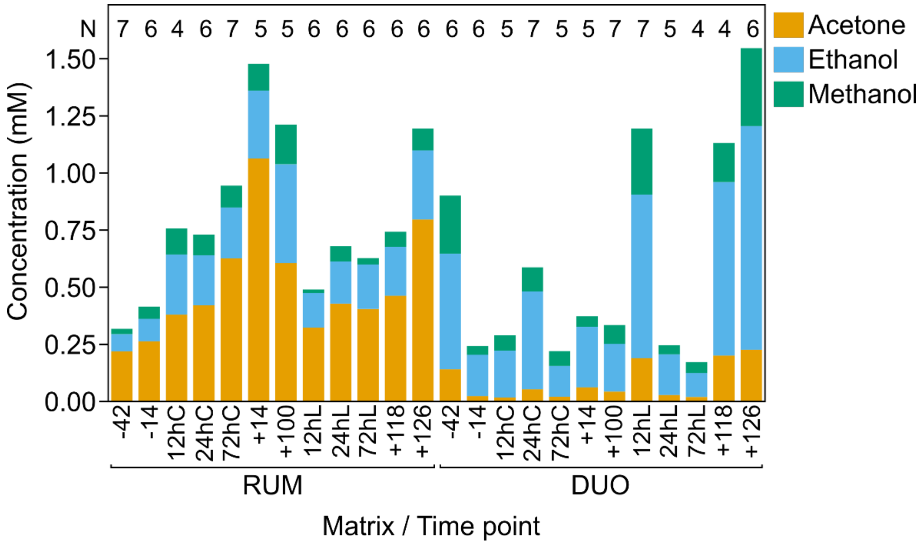

Supplementary Figure 10 Acetone, ethanol and methanol concentrations (mM) across the trial phase in rumen (RUM) and duodenal fluid samples (DUO). N refers to sample number included per time point. Time points including a "–" or "+" indicate days *antepartum* or *postpartum* and time points including "hC" or "hL" are samples taken at 12, 24 or 72 hours after calving or LPS challenge, respectively.

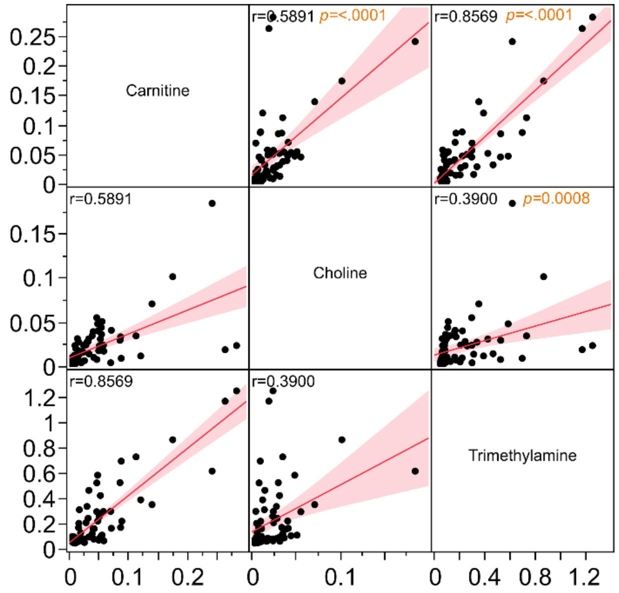

A

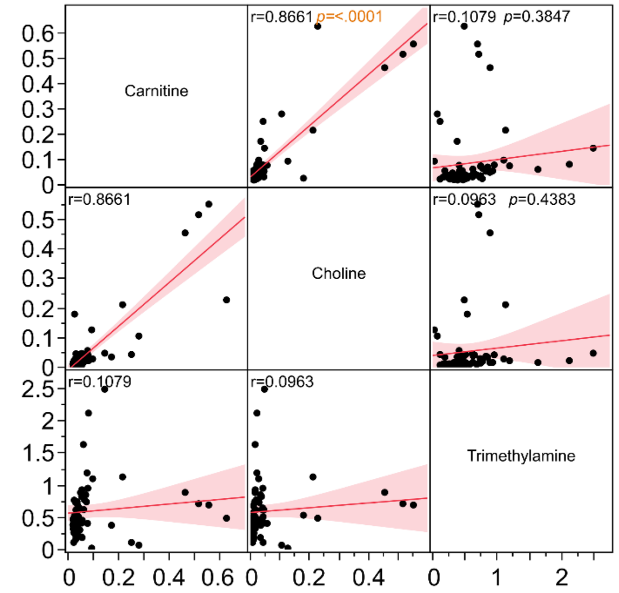

B

	Rumen			Duodenum		
	Choline	Trimethylamine	Carnitine	Choline	Trimethylamine	Carnitine
Mean	0.022	0.220	0.045	0.055	0.598	0.087
Std Dev	0.026	0.251	0.058	0.108	0.417	0.127
Std Err Mean	0.003	0.030	0.007	0.013	0.051	0.015
Upper 95% Mean	0.029	0.280	0.059	0.081	0.700	0.117
Lower 95% Mean	0.016	0.161	0.031	0.029	0.496	0.056
N	71	71	71	67	67	67
Variance	0.001	0.063	0.003	0.012	0.174	0.016

Supplementary Figure 11 Correlation scatterplot of choline, L-carnitine and trimethylamine in rumen **(A)** and duodenal fluid samples **(B)** with summary statistics.

2.2 Supplementary Tables

Supplementary Table 1 Pairwise test of rumen and duodenal fluid (N=75) samples, control (CON: N-DUO=32; N-RUM=32) and carnitine supplemented (CAR: N-DUO=43; N-RUM=36) group at same time points on OTU level (ANOSIM pairwise test; permutations=9999). At 12hC there were only 2 rumen samples available, which is why there are no values. Time points including a "–" or "+" indicate days antepartum or postpartum and time points including "hC" or "hL" are samples taken at 12, 24 or 72 hours after calving or LPS challenge, respectively.

		**Rumen**				**Duodenum**			
CON	CAR	Global-R	*p*-value	N-CON	N-CAR	Global-R	*p*-value	N-CON	N-CAR
-42	-42	-0.219	0.943	4	4	0.156	0.257	4	4
-14	-14	-0.286	0.867	4	2	0.019	0.457	4	3
12hC	12hC	-	-	2	-	0.750	0.100	2	3
24hC	24hC	0.111	0.257	3	4	0.204	0.171	3	4
72hC	72hC	0.750	0.067	2	4	0.333	0.300	2	3
+14	+14	-0.417	0.900	3	2	0.000	0.500	3	2
+100	+100	-0.500	1.000	3	2	-0.185	0.771	3	4
12hL	12hL	0.000	0.600	3	3	0.056	0.343	3	4
24hL	24hL	-0.417	1.000	2	3	-0.321	0.800	2	4
72hL	72hL	0.000	0.333	2	4	-0.214	0.800	2	4
+118	+118	0.071	0.400	2	4	-0.464	1.000	2	4
+126	+126	0.179	0.333	2	4	0.036	0.467	2	4

Supplementary Table 2 Pairwise test of rumen (RUM; N=68) and duodenal fluid (DUO; N= 75) samples on OTU level and different time points (ANOSIM pairwise test; permutations=9999). SIMPER analysis shows the main contributors to the difference with higher abundance in duodenum samples (orange) and rumen fluid samples (green). Time points including a "–" or "+" indicate days antepartum or postpartum and time points including "hC" or "hL" are samples taken at 12, 24 or 72 hours after calving or LPS challenge, respectively.

**RUM**	**DUO**	**Global-R**	***p*-value**	**SIMPER**
-42	-42	0.277	0.018 → significant	***Ruminobacter* OTU104**, ***Fibrobacter* OTU248**, ***Succiniclasticum* OTU38**
-14	-14	0.284	0.037 → significant	***Succiniclasticum* OTU38**, **uncl. Bacteroidales OTU805**, **uncl. Gammaproteobacteria OTU241**
12hC	12hC	0.273	0.238	not significant
24hC	24hC	0.010	0.374	
72hC	72hC	-0.005	0.487	
+14	+14	-0.076	0.635	
+100	+100	0.071	0.246	
12hL	12hL	0.078	0.196	
24hL	24hL	0.056	0.286	
72hL	72hL	0.002	0.437	
+118	+118	0.072	0.223	
+126	+126	-0.013	0.491	

Supplementary Table 3 Multivariate correlations (Pearson product-moment correlation coefficient) between genera and the Shannon diversity index (α-diversity), across all time points in rumen (N=68) and duodenal fluid (N=75) samples. Example: Olsenella was highly negatively correlating with high microbial diversities in rumen and duodenal fluid samples.

**Rumen**		
**Genus vs. Shannon diversity index**	**r-value**	***p*-value**
Olsenella	-0.6304	<.0001
Uncl. Lachnospiraceae	-0.4033	0.0006
Roseburia	-0.4229	0.0003
Pseudoscardovia	-0.4582	<.0001
Catenisphaera	-0.3129	0.0094
Denitrobacterium	-0.3416	0.0044
Uncl. Firmicutes	-0.3600	0.0026
Uncl. Veillonellaceae	-0.3847	0.0012
Succinivibrio	0.3139	0.0091
Uncl. Proteobacteria	0.3586	0.0027
Uncl. Subdivision5_genera_incertae_sedis	0.3674	0.0021
Uncl. Bacteroidetes	0.3714	0.0018
SR1_genera_incertae_sedis	0.3754	0.0016
Uncl. Bacteria	0.4179	0.0004
Anaeroplasma	0.4583	<.0001
Anaerovibrio	0.4618	<.0001
Uncl. Anaeroplasmataceae	0.4646	<.0001
Uncl. Spirochaetales	0.4857	<.0001
Treponema	0.5322	<.0001
Uncl. Bacteroidales	0.6679	<.0001
**Duodenum**		
**Genus vs. Shannon diversity index**	**r-value**	***p*-value**
Olsenella	-0.6146	<.0001
Uncl. Lachnospiraceae	-0.5066	<.0001
Roseburia	-0.3943	0.0005
Acidaminococcus	-0.3534	0.0019
Allisonella	-0.3051	0.0078
Bacteroides	-0.3102	0.0068
Uncl. Enterobacteriaceae	-0.3163	0.0057

Clostridium sensu stricto	-0.3180	0.0054
Enterococcus	-0.3171	0.0056
Peptostreptococcus	-0.3133	0.0062
Romboutsia	-0.3171	0.0056
Uncl. Clostridiales_Incertae Sedis XI	-0.3150	0.0059
Uncl. Coriobacteriales	-0.3336	0.0035
Uncl. Firmicutes	-0.3695	0.0011
Uncl. Peptostreptococcaceae	-0.3171	0.0056
Uncl. Selenomonadales	-0.3863	0.0006
Uncl. Anaeroplasmataceae	0.3668	0.0012
Ruminobacter	0.3557	0.0017
Succinivibrio	0.3359	0.0032
Uncl. Proteobacteria	0.3647	0.0013
Fibrobacter	0.3613	0.0014
SR1_genera_incertae_sedis	0.3188	0.0053
Uncl. Spirochaetaceae	0.3294	0.0039
Uncl. Spirochaetales	0.3775	0.0008
Uncl. Betaproteobacteria	0.4412	<.0001
Uncl. Burkholderiales	0.4290	0.0001
Uncl. Bacteroidia	0.3940	0.0005
Uncl. Ruminococcaceae	0.4298	0.0001
Anaeroplasma	0.5219	<.0001
Treponema	0.4992	<.0001
Uncl. Bacteroidetes	0.5157	<.0001
Uncl. Bacteroidales	0.7004	<.0001

Supplementary Table 4 Average concentrations of metabolites across all 12 time points significantly (non-parametric Wilcoxon test $p \leq 0.005$) higher in rumen (green, N=71) and duodenum samples (orange, N=67).

	**Rumen** (N=71)		**Duodenum** (N=67)	
	Average (mM)	SD	Average (mM)	SD
**Acetate**	**74.01**	14.62	**1.38**	3.82
**Propionate**	**27.43**	10.39	**0.88**	0.15
**Butyrate**	**12.40**	3.49	**0.05**	0.98
**Adipate**	**3.02**	0.85	**0.43**	0.67
**Pimelate**	**2.59**	1.13	**0.38**	0.63
**Valerate**	**3.39**	1.57	**0.74**	1.22
**Acetone**	**0.50**	0.36	**0.09**	0.19
**Isobutyrate**	**1.31**	0.96	**0.42**	0.45
**Imidazole**	**0.32**	0.13	**0.29**	1.01
**Dimethylamine**	**0.04**	0.06	**1.60**	0.67
**Cadaverine**	**0.06**	0.11	**0.63**	0.61
**Trimethylamine**	**0.22**	0.25	**0.60**	0.42
**Leucine**	**0.21**	0.14	**0.67**	0.69
**Ornithine**	**0.15**	0.13	**0.72**	0.93
**3-Hydroxybutyrate**	**0.13**	0.09	**0.46**	0.55
**Aspartate**	**0.14**	0.29	**2.05**	3.30
**Glutamate**	**0.25**	0.20	**1.36**	2.23
**Methylamine**	**0.25**	0.22	**0.53**	0.52
**Phenylacetate**	**0.21**	0.14	**0.56**	0.69
**Lysine**	**0.15**	0.22	**0.71**	1.16
**Isopropanol**	**0.11**	0.07	**0.23**	0.24
**2-Phenylpropionate**	**0.19**	0.09	**0.40**	0.52
**Phenylalanine**	**0.07**	0.05	**0.46**	1.02
**Glycine**	**0.22**	0.27	**1.80**	4.15
**Allantoin**	**1.85**	3.00	**3.36**	3.11
**Carnitine**	**0.05**	0.06	**0.09**	0.13
**Hypoxanthine**	**0.12**	0.11	**0.25**	0.93
**Formate**	**0.24**	0.19	**0.33**	1.05
**3-Phenylpropionate**	**0.49**	0.10	**0.51**	0.94
**Glucose**	**0.70**	0.93	**0.73**	2.29

Supplementary Table 5 Correlation coefficients (r≥0.3) between the family Coriobacteriaceae and rumen (N=66) and duodenal fluid (N=67) metabolites across all time points.

Rumen fluid			Duodenum fluid		
**Metabolite**	**r-value**	***p*-value**	**Metabolite**	**r-value**	***p*-value**
Valerate	0.6186	<.0001	Glycine	0.7109	<.0001
Pimelate	0.5579	<.0001	Propionate	0.5115	<.0001
4-Hydroxybutyrate	0.5164	<.0001	Isobutyrate	0.4715	<.0001
Leucine	0.5120	<.0001	Leucine	0.4642	<.0001
Alanine	0.5081	<.0001	Valerate	0.4634	<.0001
Isopropanol	0.5074	<.0001	Carnitine	0.4254	0.0003
Acetone	0.5014	<.0001	Butyrate	0.4128	0.0005
Carnitine	0.4810	<.0001	Isoleucine	0.4008	0.0008
Propionate	0.4727	<.0001	Acetoacetate	0.3943	0.0010
Valine	0.4228	0.0004	Pimelate	0.3915	0.0011
Acetoacetate	0.3773	0.0018	Isovalerate	0.3693	0.0021
Isoleucine	0.3729	0.0020	Alanine	0.2995	0.0138
Glycine	0.3718	0.0021			
Lysine	0.3612	0.0029			
Isovalerate	0.3065	0.0123			
Glutamate	0.3004	0.0143			
Phenylalanine	0.2944	0.0164			

Supplementary Table 6 Shared and unique rumen (N=66) and duodenal (N=67) genera which, according to correlation r-values and corresponding p-values may be involved in carnitine metabolism.

**Shared sig. carnitine influenced taxa on genus-level**	**Rumen**		**Duodenum**	
	**r-value**	***p*-value**	**r-value**	***p*-value**
Uncl. Bacteroidales	-0.3880	0.0013	-0.3154	0.0093
Campylobacter	-0.1736	0.1632	0.5408	<.0001
Uncl. Actinomycetaceae	-0.1017	0.4164	0.5100	<.0001
Uncl. Firmicutes	0.2884	0.0188	0.4311	0.0003
Denitrobacterium	0.3431	0.0048	0.2818	0.0209
Uncl. Selenomonadales	0.4128	0.0006	0.0456	0.7143
Uncl. Veillonellaceae	0.5005	<.0001	-0.0826	0.5065
Olsenella	0.5029	<.0001	0.4237	0.0004
Pseudoscardovia	0.6124	<.0001	0.1833	0.1375

**Rumen unique carnitine influenced genera**	**r-value**	***p*-value**
Uncl. Bacteria	-0.3191	0.0090
Uncl. Proteobacteria	-0.2719	0.0272
Treponema	-0.2550	0.0388
Uncl. Lachnospiraceae	0.2385	0.0538
Syntrophococcus	0.4143	0.0005
Roseburia	0.4899	<.0001

**Duodenum unique carnitine influenced genera**	**r-value**	***p*-value**
Uncl. Eubacteriaceae	0.2473	0.0437
Uncl. Succinivibrionaceae	0.3511	0.0036

Supplementary Table 7 Correlation table including *Olsenella*, *Pseudoscardovia* and uncl. Veillonellaceae and the metabolites carnitine, trimethylamine and choline. Orange + indicate significant (p≤0.05) positive and red – significant negative correlations. Bold highlighted RUM (rumen, N=66) and DUO (duodenum, N=67) indicate dominant site for the specific significant combination. Time points including a "–" or "+" indicate days antepartum or postpartum and time points including "hC" or "hL" are samples taken at 12, 24 or 72 hours after calving or LPS challenge, respectively.

Combination	*Olsenella* - Choline		*Olsenella* - Carnitine		*Olsenella* - Trimethylamine		*Pseudoscardovia* - Trimethylamine		*Pseudoscardovia* - Choline		*Pseudoscardovia* - Carnitine		*Uncl. Veillonellaceae* - Trimethylamine		*Uncl. Veillonellaceae* - Carnitine	
-42																
-14				+		+										
12hC		+												-		
24hC							+		+		+					
72hC																-
+14				+								+			+	
+100		+		+		+			+							
12hL					-			-								
24hL								+					+			
72hL																
+118								-	+							
+126																
Matrix	RUM	DUO	RUM	DUO	RUM	DUO	RUM	DUO	RUM	DUO	RUM	DUO	RUM	DUO	RUM	DUO
Count	2		3		3		4		3		2		2		2	
